# High mammographic density is associated with an increase in stromal collagen and immune cells within the mammary epithelium

**DOI:** 10.1186/s13058-015-0592-1

**Published:** 2015-06-04

**Authors:** Cecilia W. Huo, Grace Chew, Prue Hill, Dexing Huang, Wendy Ingman, Leigh Hodson, Kristy A. Brown, Astrid Magenau, Amr H. Allam, Ewan McGhee, Paul Timpson, Michael A. Henderson, Erik W. Thompson, Kara Britt

**Affiliations:** University of Melbourne Department of Surgery, St. Vincent’s Hospital, Level 2, Clinical Sciences Building, 29 Regent Street, Fitzroy, VIC 3065 Australia; Department of Pathology, St. Vincent’s Hospital, 41 Victoria Parade, Fitzroy, VIC 3065 Australia; St. Vincent’s Institute, 9 Princes Street, Fitzroy, VIC 3065 Australia; Discipline of Surgery, Faculty of Health Sciences, School of Medicine, The Queen Elizabeth Hospital, University of Adelaide, Adelaide, Australia; Robinson Research Institute, University of Adelaide, Ground Floor, Norwich Centre, 55 King William Road, North Adelaide, SA 5006 Australia; Hudson Institute of Medical Research, 27-31 Wright Street, Clayton, VIC 3168 Australia; Garvan Institute of Medical Research, 384 Victoria Street, Darlinghurst, Australia; St Vincent’s Clinical School, Faculty of Medicine, University of NSW, Sydney, Australia; The Beatson Institute for Cancer Research, Switchback Road, Bearsden Glasgow, G61 1BD UK; Peter MacCallum Cancer Centre, 2 St. Andrews Place, East Melbourne, VIC 3002 Australia; Institute of Health and Biomedical Innovation and School of Biomedical Sciences, Queensland University of Technology, 60 Musk Avenue, Kelvin Grove, QLD 4059 Australia; The Sir Peter MacCallum Department of Oncology, University of Melbourne, St. Andrews Place, East Melbourne, VIC 3002 Australia; Department of Anatomy and Developmental Biology, Monash University, 19 Innovation Walk, Clayton, VIC s Australia; St Vincent’s Clinical School, Faculty of Medicine, University of NSW, Clayton, Australia

## Abstract

**Introduction:**

Mammographic density (MD), after adjustment for a women’s age and body mass index, is a strong and independent risk factor for breast cancer (BC). Although the BC risk attributable to increased MD is significant in healthy women, the biological basis of high mammographic density (HMD) causation and how it raises BC risk remain elusive. We assessed the histological and immunohistochemical differences between matched HMD and low mammographic density (LMD) breast tissues from healthy women to define which cell features may mediate the increased MD and MD-associated BC risk.

**Methods:**

Tissues were obtained between 2008 and 2013 from 41 women undergoing prophylactic mastectomy because of their high BC risk profile. Tissue slices resected from the mastectomy specimens were X-rayed, then HMD and LMD regions were dissected based on radiological appearance. The histological composition, aromatase immunoreactivity, hormone receptor status and proliferation status were assessed, as were collagen amount and orientation, epithelial subsets and immune cell status.

**Results:**

HMD tissue had a significantly greater proportion of stroma, collagen and epithelium, as well as less fat, than LMD tissue did. Second harmonic generation imaging demonstrated more organised stromal collagen in HMD tissues than in LMD tissues. There was significantly more aromatase immunoreactivity in both the stromal and glandular regions of HMD tissues than in those regions of LMD tissues, although no significant differences in levels of oestrogen receptor, progesterone receptor or Ki-67 expression were detected. The number of macrophages within the epithelium or stroma did not change; however, HMD stroma exhibited less CD206^+^ alternatively activated macrophages. Epithelial cell maturation was not altered in HMD samples, and no evidence of epithelial–mesenchymal transition was seen; however, there was a significant increase in vimentin^+^/CD45^+^ immune cells within the epithelial layer in HMD tissues.

**Conclusions:**

We confirmed increased proportions of stroma and epithelium, increased aromatase activity and no changes in hormone receptor or Ki-67 marker status in HMD tissue. The HMD region showed increased collagen deposition and organisation as well as decreased alternatively activated macrophages in the stroma. The HMD epithelium may be a site for local inflammation, as we observed a significant increase in CD45^+^/vimentin^+^ immune cells in this area.

**Electronic supplementary material:**

The online version of this article (doi:10.1186/s13058-015-0592-1) contains supplementary material, which is available to authorized users.

## Introduction

Important risk factors for breast cancer (BC) are increasing age, genetic mutations, family history or personal history of BC and mammographic density (MD), which refers to the extent of opaque appearance on a mammogram [1]. The radiological appearance of the breast differs among women owing to varying proportions of radiodense fibroglandular tissue that appears white and radiolucent adipose tissue that appears dark [2]. Women with breasts that have 75 % or greater mammographically dense tissue are four to six times more likely to develop BC than are women with breasts that have 10 % or less mammographically dense tissue, and this risk has been shown to persist over 10 years of follow-up [3].

High mammographic density (HMD) is not uncommon in the community. In a study of 1353 women in America, researchers found that 42 % of the 40- to 59-year-old age group and 25 % of the 60- to 79-year old age group have breasts that are at least 50 % mammographically dense [4]. Although 55 % to 65 % of *BRCA1* mutation carriers develop BC by the age of 70, these cases are estimated to account for just 2 % to 5 % of all BCs [5, 6]. By contrast, the prevalence of HMD suggests that as many as 30 % of BC cases could be attributable to elevated MD [7], should the MD–BC risk association be causal [8]. The link between high MD and BC risk is now in the public domain. Advocacy groups such as *Are You Dense?* [9] have provided education to the public and helped advocate changes in public policy. As of December 2014, Breast Density Inform laws, which call for mandatory reporting of MD status, had been introduced in 20 states across the United States [10]. As the biological basis of MD is not understood, viable preventative and/or therapeutic targets aimed at reducing MD-associated BC risk have yet to be developed.

To date, various studies have examined the histological and molecular differences of HMD and low mammographic density (LMD) breast tissues. HMD tissue has been shown to be composed of greater areas of stroma, but less fat, than LMD tissue; however, findings on whether HMD area contains higher amounts of epithelium are conflicting [11, 12], and epithelial cell proliferation appeared to be increased in some studies, but not in others [11, 13]. The proportion of basal and luminal cell layers within the epithelium has never been assessed, and change in this proportion is one of the first steps in tumorigenesis [14, 15]. The composition of the stroma has been examined in a few studies [12, 16, 17], which have shown that the degree of collagen deposition is increased in HMD tissue [18–20]; however, collagen organisation has not been assessed. The abundance and phenotype of immune cells, now an acknowledged component of the tumour microenvironment [21], have also never been assessed in HMD tissue.

In an attempt to define the tissue components responsible for the increased MD, we analysed the epithelial, stromal and adipose compositions and further investigated the stromal and epithelial compartments. We assessed differentiation, proliferation, hormone receptor and aromatase expression in the epithelium; collagen content and organisation; and immune cell status. In an effort to assess the possible role of epithelial–mesenchymal transition (EMT), which has been implicated in many aspects of malignancy [22, 23], we included vimentin in our analysis of epithelial cells.

## Methods

### The study population

This study was approved by the Peter MacCallum Human Research Ethics Committee (number 08/21) and St. Vincent’s Hospital Animal Ethics Committee (number 049/09). It was conducted in accordance with the Australian National Statement on Ethical Conduct in Human Research [24]. Between 2008 and 2013, 48 women undergoing prophylactic mastectomy for BC prevention at St. Vincent’s Hospital were consented through the Victorian Cancer Biobank (VCB 10010) to participate in this study. These women had either a confirmed *BRCA1/2* carrier status, a past history of BC in the other breast or a family history of two or more first- or second-degree relatives with BC who were diagnosed before the age of 50 years. Women were excluded from this study if there were any clinical suspicions of ductal carcinoma in situ (DCIS) or microcalcifications seen on radiological investigations.

### Tissue accrual and selection of high and low mammographically dense regions

Resected breast tissue was transferred immediately to the pathology department upon completion of mastectomy, where pathologists resected slices of breast tissue using a sterile technique. X-rays of those slices were taken by a breast radiographer using uniform radiological parameters and were assessed against a calibration ruler for selection of HMD and LMD regions. The tissue slice was transferred to a biosafety level 2 hood, where areas that appeared white on X-rays were selected and removed using sterile blades and defined as HMD regions, and areas that appeared black were similarly selected, removed and classified as LMD regions. When the tissue slice mammogram appeared homogeneously grey or lacked black-white differential, the patient was excluded from the study. This method was detailed in our previous work [12, 25].

The tissue samples were then fixed in 10 % neutral buffered formalin, processed through alcohol, embedded in paraffin blocks and then sectioned at 5-μm thickness for histological analyses.

### Haematoxylin and eosin staining

Conventional haematoxylin and eosin (H&E) staining was performed on paired HMD and LMD tissue sections cut from the blocks as described above. Stained entire tissue sections were photographed using an Aperio ScanScope XT digital microscope (Leica Biosystems, Buffalo Grove, IL, USA) at ×20 magnification to generate high-resolution images, which were then exported into JMicroVision image analysis software (v.1.2.7; University of Geneva, Geneva, Switzerland). The whole tissue image was used to assess tissue composition, which was achieved by manually demarcating the entire tissue area as well as individual areas of dense connective tissue and epithelium and applying thresholding masks to stromal and epithelial areas separately to calculate the percentage of these components relative to the whole tissue section. This method has been described in detail previously [25].

### Mammographic density tissue index

To assess whether a semiquantitative histological assessment of MD could help select HMD and LMD specimens where radiological guidance may be unavailable, an MD tissue index was employed to examine any potential difference between HMD and LMD tissues that were sampled, based on their X-ray appearance, in this study. Two examiners were independently blinded to all details of the specimen and, by looking at the whole tissue section, assigned the tissue samples to one of the following five categories: (1) 0 to 10 % of the tissue section was stroma and/or epithelium; (2) 11 % to 25 % of the tissue section was stroma and/or epithelium; (3) 26 % to 50 % of the tissue section was stroma and/or epithelium; (4) 51 % to 75 % of the tissue section was stroma and/or epithelium; or (5) more than 75 % of the tissue section was stroma and/or epithelium.

### Immunohistochemical Masson’s trichrome blue staining

A total of 15 pairs of HMD and LMD tissue samples were selected, based on the presence of epithelium on H&E-stained images, to represent the characteristics of the mammary specimens. They were used for all further analyses. Masson’s trichrome blue (MTB) staining was used to demonstrate the quantity of fibrillar collagens in HMD and LMD tissue sections, and Ki-67 antibody (clone MIB-1, catalogue number M7240; Dako, Carpinteria, CA, USA) was used for assessing and comparing proliferation status. Oestrogen receptor (ER, clone SP1, catalogue number 790-2223; Ventana Medical Systems, Tucson, AZ, USA) and progesterone receptor (PR, clone SP1, catalogue number 790-2223; Ventana Medical Systems) status were also analysed immunohistochemically. Staining was performed as described previously [25]. JMicroVision image analysis software v.1.2.7 was used to quantify nuclear staining of ER, PR and Ki-67. Glandular areas were manually selected as polygons, and positive and negative staining was assigned as class 1 and 2, respectively, with different keystrokes. The point-counting system was used to randomly count approximately 300 points for calculation of the percentage of positive staining in total glandular areas per whole tissue section. Similarly to H&E-stained tissue sections, MTB-stained tissue slides were scanned using an Aperio digital microscope, and high-resolution images were exported into JMicroVision for quantification of collagen content by thresholding the blue-stained collagen and calculating the percentage of collagen per whole tissue section.

### Aromatase immunostaining

Aromatase immunostaining was performed as previously described [26]. In brief, tissue sections were incubated in 10 % horse serum in CAS-Block reagent (Invitrogen/Life Technologies, Carlsbad, CA, USA) for 30 minutes. Previously characterized aromatase mouse monoclonal primary antibody 677 (1:750 dilution from 2.6 mg/ml stock in 0.5 % bovine serum albumin/phosphate-buffered saline (PBS) [27]) was added to the slides and incubated overnight at 4 °C. After the slides were washed in PBS, biotinylated universal secondary antibody (1:200 dilution, VECTASTAIN Elite ABC Kit (Mouse IgG); Vector Laboratories, Burlingame, CA, USA) was applied for 30 minutes and then incubated with VECTASTAIN ABC-HP reagent for 30 minutes. 3,3-Diaminobenzidine tetrahydrochloride (Sigma-Aldrich, St. Louis, MO, USA) was added until the desired cytoplasmic brown stain intensity developed, and the reaction was then stopped with distilled water. For scoring, we used four high-magnification (×40) images of each section of HMD and LMD obtained from each of the 15 women. Sections were blinded for assessment, and two independent examiners determined the levels of stromal and epithelial aromatase expression as proportions of total epithelial and stromal cells.

### Second harmonic generation imaging

Unstained paraffin sections (15 μm) of HMD and LMD tissue obtained from eight randomly selected women were used for second harmonic generation (SHG) imaging. Imaging was performed on a Leica SP8 inverted multiphoton microscope (Leica Microsystems) with a long working distance, ×25 magnification, 0.95 numerical aperture water objective. The multiphoton laser (Chameleon Vision II; Coherent, Santa Clara, CA, USA) was operated at 80 MHz and tuned to an excitation wavelength of 960 nm. SHG signals were collected on a nondescanned external hybrid detector located at the back focal plane of the objective. A 480/40-nm emission filter was used, and 20-μm z-stacks with 2.5-μm step sizes were generated for each section.

### Grey-level co-occurrence matrix analysis

Grey-level co-occurrence matrix (GLCM) analysis was performed to assess stromal collagen fibre structure and organisation in HMD and LMD tissue samples. This method allows assessment of the level of structural collagen organisation, as previously described [28, 29]. GLCM was performed using a macro in ImageJ software (National Institutes of Health, Bethesda, MD, USA). First, the user selected a directory containing the collagen stack images. A maximum projection image was then produced and duplicated, from which the user selected three 74 × 74–μm regions of interest (ROIs). The image data in each ROI was passed to the GLCM plugin (UMB GLCM features [30]), which was modified to allow the testing of numerous directions and distances of comparison. The output of the plugin for each ROI was 100 rows of the 5 texture parameters (contrast, uniformity, correlation, homogeneity and entropy) over each of 4 directions, so, in total, 2000 parameter values were obtained. These were saved as a text data file for each ROI. Once all the images in the directory were analysed, the data files were processed using a MATLAB (MathWorks, Chatswood, Australia) script that outputs the mean (with SEM) value of each texture parameter for each image. The results of the GLCM correlation where then imported into GraphPad, Prism software (GraphPad Software, La Jolla, CA, USA), where a double exponential decay model was fitted to the data and the weighted mean decay distance for each sample was calculated.

### Macrophage staining

To assess the effect of MD on the abundance, location and phenotype of macrophages, the 15 paired LMD and HMD tissue sections were stained with a pan-macrophage marker CD68 (clone PG-M1, catalogue number M0876; Dako), as well as CD206, also known as macrophage mannose receptor 1, which is a marker of alternative macrophage activation involved in resolution of inflammation (CD206 monoclonal antibody, clone 685645; R&D Systems, Minneapolis, MN, USA). The stained sections were imaged using a NanoZoomer digital slide scanner (Hamamatsu Photonics, Hamamatsu, Japan). Epithelial cell– and stromal cell–associated macrophages were manually counted and quantified per square millimetre of tissue in three randomly selected glandular and stromal areas within each tissue sample.

### Immunofluorescence staining for lineage markers

Cytokeratin-19 [CK-19, Pierce A53-B/A2.26 (Ks19.1); Thermo Scientific, Waltham, MA, USA], CK-14 (CBL197; EMD Millipore, Bayswater, Australia) and vimentin (clone V9, catalogue number M0725; Dako) triple immunofluorescence (IF) staining was performed on the selected 15 pairs of HMD and LMD tissues. CK-19 and CK-14 were used to delineate luminal epithelial cells and basal epithelial cells, respectively. To enhance the clarity of histological composition, vimentin was also used to illustrate the stromal component in both HMD and LMD groups, as well as any evidence of EMT. Five images of glandular areas were taken randomly for each specimen using a Nikon C2si confocal microscope (Nikon Instruments, Melville, NY, USA). Point counting using JMicroVision, similarly to that employed for ER, PR and Ki-67 quantitative analyses as mentioned above, was used to assess the proportions of basal epithelial cells stained for CK-14, luminal epithelial cells stained for CK-19, and mesenchymal cells stained for vimentin. Triple staining was performed to examine the level of immune cell infiltration in these tissues, pan-macrophage marker CD68 (clone PG-M1, catalogue number M0876; Dako), pan-CK (clone Oscar, catalogue number MON3267; MONO*SAN*, Uden, the Netherlands) and mesenchymal marker vimentin (clone V9; Dako). Furthermore, pan-inflammatory marker CD45 (clones 2B11 and pd7/26, catalogue number M0701; Dako) and mesenchymal marker vimentin (clone LN-6, catalogue number V2258; Sigma-Aldrich) double IF staining was performed.

### Statistical analysis

HMD and LMD tissue biopsies from the same breast of the same woman were analysed in a pairwise manner, which overcame potential confounding factors such as age, body mass index (BMI), menstrual cycle and metabolic activity. Normality was determined using the D’Agostino-Pearson omnibus normality test. Wilcoxon matched-pairs signed-rank test was used for nonparametric data to determine the percentage composition of epithelial, stromal and adipose tissues within each sample for H&E-stained tissue; of collagen for MTB-stained tissue; for macrophage staining; and of basal, luminal and mesenchymal cells in CK- and vimentin-stained tissues. For parametric data, a paired *t* test was used to compare the percentage of ER and PR staining and of Ki-67 expression between HMD and LMD tissues. The *P* value was required to be less than 0.05 to achieve statistical significance. All tests were performed in GraphPad, Prism software.

## Results

### The study cohort

A total of 48 high-risk women were included in this study; however, of these, 4 women had suspicious DCIS based on specimen radiographs, and 3 women had tissue samples with a homogeneous radiological appearance. Thus, these 7 women were excluded, and their specimens were sent for standard pathological processing, leaving a final cohort of 41 women eligible for this study.

The participant characteristics are detailed in Table 1. All candidates were Caucasian, and their mean age was 43 years (range 31-59 years). They all underwent prophylactic mastectomy owing to a high BC risk profile. Of the 41 participants, 28 were premenopausal and 37 had borne children. Their MD was rated according to the BI-RADS classification scheme. Their final histopathology reports regarding their mastectomy tissue confirmed that the breast was free of DCIS or cancer. Of the 15 women selected for further analysis, 14 were premenopausal and 13 had borne children. Hence, our overall results represented a premenopausal and parous cohort.

**Table 1:** Demographic characteristics of study participants

Selected characteristics		Number or mean
Age at surgery		Mean 43 yr (range 31–59 yr)
BI-RADS category		
	4	11
	3	16
	2	10
	1	4
Risk factors		
	*BRCA*−, strong family history	9
	*BRCA1*+	15
	*BRCA2*+	13
	Past history of BC or DCIS	24
Menopausal status		
	Premenopausal	28
	Perimenopausal	5
	Postmenopausal	8
Parity		
	Parous	37
	Nulliparous	4

BI-RADS score 1: predominantly fat, 2: scattered fibroglandular densities, 3: heterogeneously dense, 4: extremely dense.

*Abbreviations*: *BC* breast cancer, *BI-RADS* Breast Imaging-Reporting and Data System, *DCIS* ductal carcinoma in situ, *IDC* invasive ductal carcinoma.

### Histological composition of high and low mammographic density tissue

**Fig. 1:**
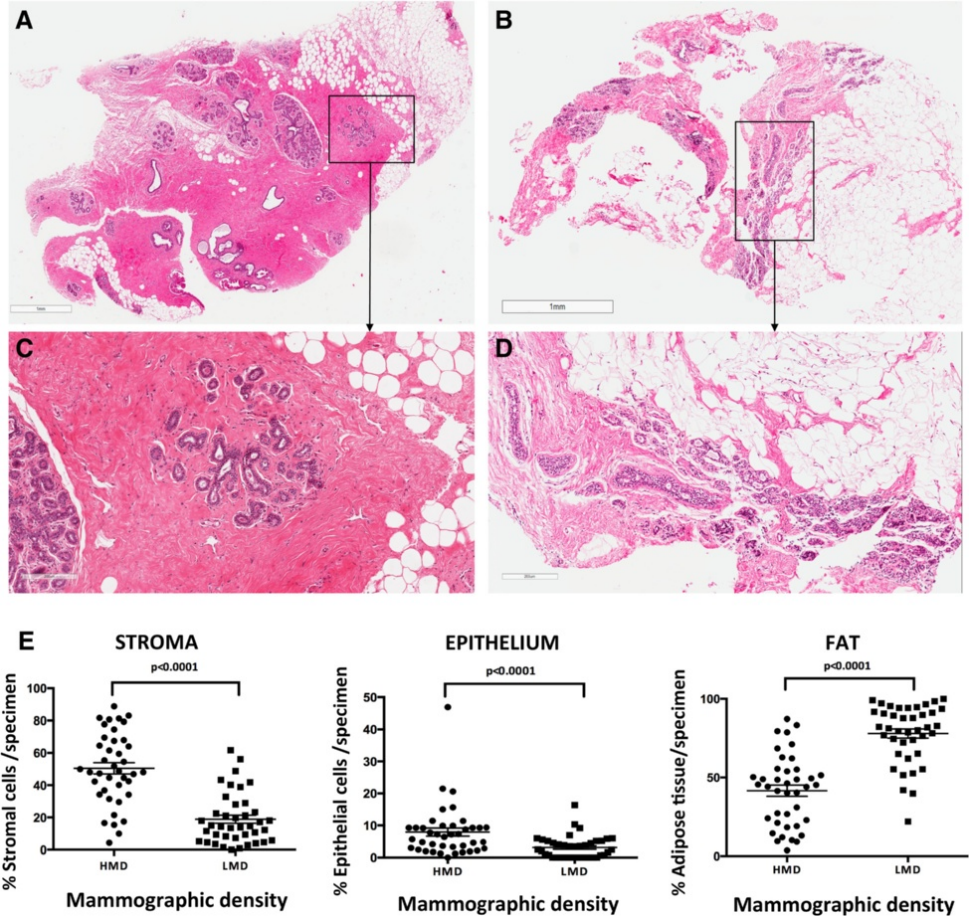
Haematoxylin and eosin–stained high mammographic density (HMD) and low mammographic density (LMD) tissue sections. (**a**) Representative photomicrograph of tissue specimen resected from an HMD region. (**b**) Representative photomicrograph of tissue specimen resected from an LMD region of the same healthy breast shown in (A). (**c**) Selected area from HMD tissue shown in (A) at ×10 original magnification. (**d**) Selected area from HMD tissue shown in (B) at ×10 original magnification. (**e**) Scatterplots of percentages of stroma, epithelium and fat of HMD and LMD within-individual tissue (N = 41 women)

Quantitative analysis of 41 paired specimens found that HMD tissue had 31.57 % more stroma, 4.82 % more glandular area and 36.39 % less fat than LMD tissue in terms of mean percentage, and each of those differences was statistically significant (*P* < 0.0001) (Fig. 1). If a semiquantitative histological MD tissue index as defined in the Methods section was used. This index was developed to assess MD in samples where mammograms are not readily available, and the HMD samples classified as such by X-ray analysis were also scored as HMD in the MD tissue index (*P* < 0.0001) (see Additional file 1). We further divided the data according to parity status (parous vs. nulliparous) and menopausal status (pre-, peri- or postmenopausal). Separate quantitative analyses by parity and menopausal status showed that the differences of epithelium, stroma and fat composition between HMD and LMD tissue were still significant in 37 parous women and in 28 premenopausal women. In a parous and premenopausal subset of 25 women, HMD tissue exhibited the same patterns of significantly increased epithelium, stroma and reduced fat compared with LMD tissue (see Additional files 2 and 3).

### Masson’s trichrome blue staining

**Fig. 2:**
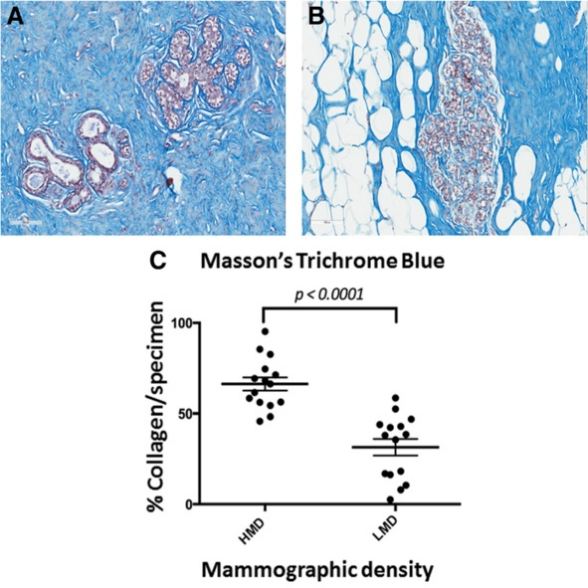
Masson’s trichrome blue staining of high mammographic density (HMD) and low mammographic density (LMD) tissue samples. Photomicrographs show representative staining patterns of HMD tissue (**a**) and LMD tissue (**b**) of the 15 women assessed. (**c**) Scatterplot shows the percentages of blue-stained collagen of HMD and LMD tissue

Consistent with the stromal differentials between HMD and LMD tissues that were evident on H&E staining, MTB staining showed a significantly higher amount of fibrillar collagen in HMD tissue than in LMD tissue (*P* < 0.0001). In both groups, collagen aligned circumferentially around glandular epithelium, although it was more densely packed in HMD sections than in LMD sections, as shown in Fig. 2.

### Second harmonic generation imaging and grey-level co-occurrence matrix analysis of stromal collagen

**Fig. 3:**
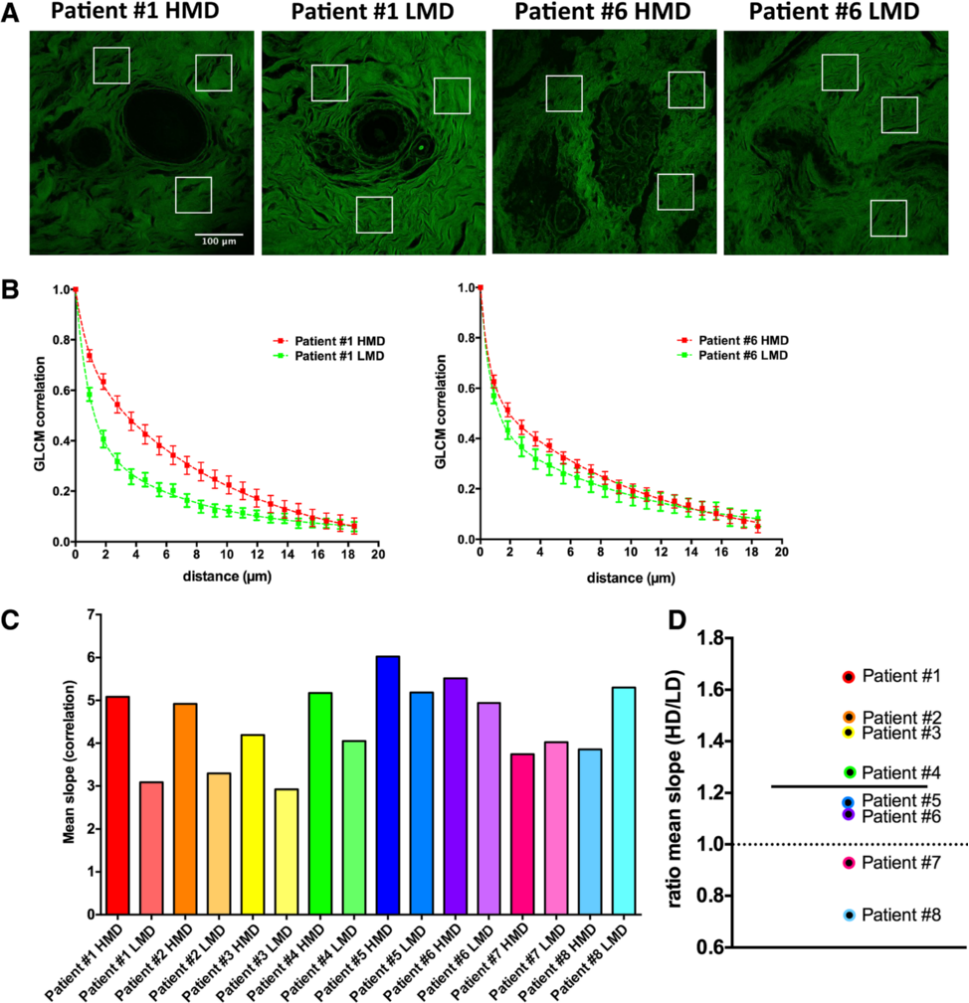
Second harmonic generation (SHG) analysis of collagen. (**a**) Collagen was imaged using an SHG technique. *White squares* indicate analysed regions. Scale bar indicates 100 μm. (**b**) Grey-level co-occurrence matrix (GLCM) analysis shows the correlation of the intensity of the SHG signal across the analysed region. Samples with a slower decay have a denser collagen structure than samples with a faster decay. For each sample, three regions were imaged. Within each region, three areas were selected for analysis (total N = 9). Mean parameter values are shown as symbols. *Dashed lines* represent the nonlinear biexponential fit to each data set. *Error bars* indicate SEM. (**c**) The mean decay parameter (slope) in a biexponential fit model of correlation decay curve was calculated and plotted for each sample. (**d**) The ratio of the mean decay of high mammographic density (HMD) to low mammographic density (LMD) was calculated to determine whether HMD samples contained a higher collagen organisation than LMD samples. A ratio above 1 indicates that HMD samples had a higher collagen organisation than LMD samples. Seventy-five percent of HMD samples showed a higher mean decay than LMD samples. *Horizontal bar* indicates the mean

On eight matched, randomly selected HMD and LMD samples, we assessed the collagen structure using SHG imaging coupled with GLCM texture analysis. The GLCM analysis shows the correlation of the intensity of the SHG signal across the analysed region. Samples with a slower decay have a more organized, uniform collagen structure than samples with a faster decay. The mean decay parameter (slope) in a biexponential fit model of correlation decay curve was calculated and plotted for each sample (matched HMD and LMD samples, indicated on the *x*-axis). The ratio of the mean decay of HMD to LMD was calculated to determine whether HMD samples contained a higher collagen organization than LMD samples. A ratio above 1 indicates that HMD samples had a higher collagen organization than LMD samples. We found that 75 % of HMD patient samples showed a higher mean decay than LMD patient samples (Fig. 3).

### Commonly used breast cancer markers in high and low mammographic density tissue

**Fig. 4:**
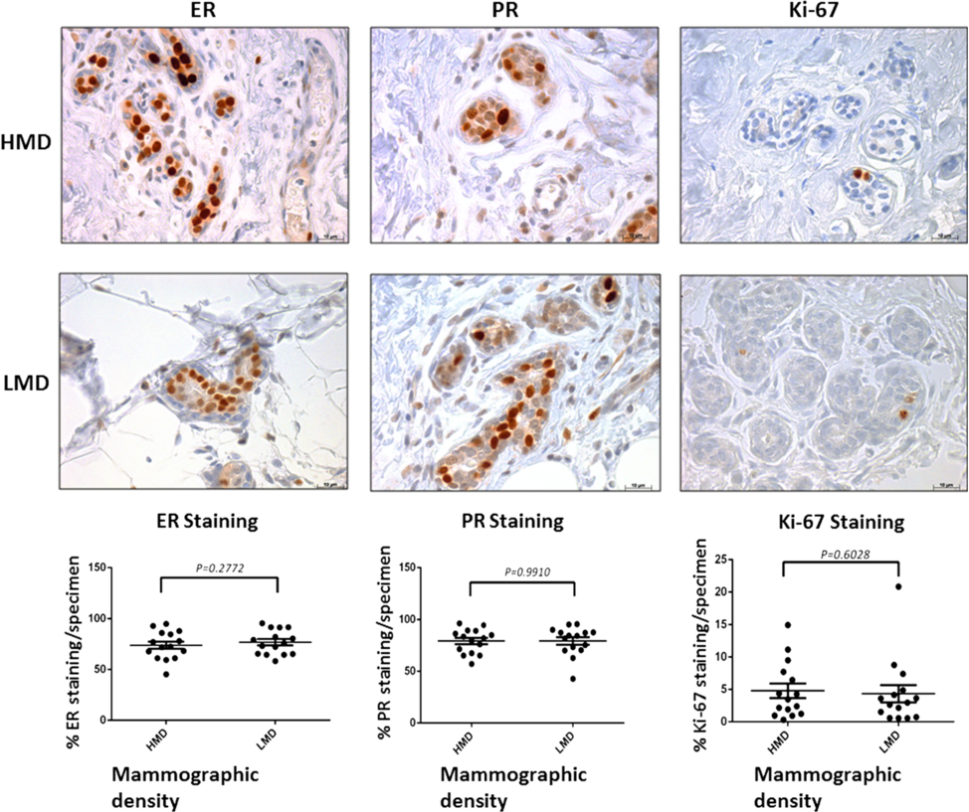
Oestrogen receptor (ER), progesterone receptor (PR) and Ki-67 staining of high mammographic density (HMD) and low mammographic density (LMD) tissue samples. Representative photomicrographs show ER, PR and Ki-67 brown nuclear staining in selected HMD and LMD regions of the 15 women. The percentages of positive nuclear staining in glandular areas per whole tissue section were point-counted using JMicroVision, and the results were analysed using paired *t* tests

ER, PR and Ki-67 staining occurred predominantly in the epithelial areas, with sparse staining observed in the stromal cells, as illustrated in Fig. 4. The percentage of ER and PR nuclear staining in glandular area per whole tissue section was similar between HMD and LMD specimens. Quantitative analyses by point counting of the glandular area confirmed that there were no statistically significant differences. Ki-67-positive expression as a marker for proliferation was overall less than 10 % of stained nuclei of all epithelial cells per tissue section in both HMD and LMD tissues. There was a nonsignificant trend for HMD tissue to have a mean 2 % higher Ki-67 expression than LMD tissue.

**Fig. 5:**
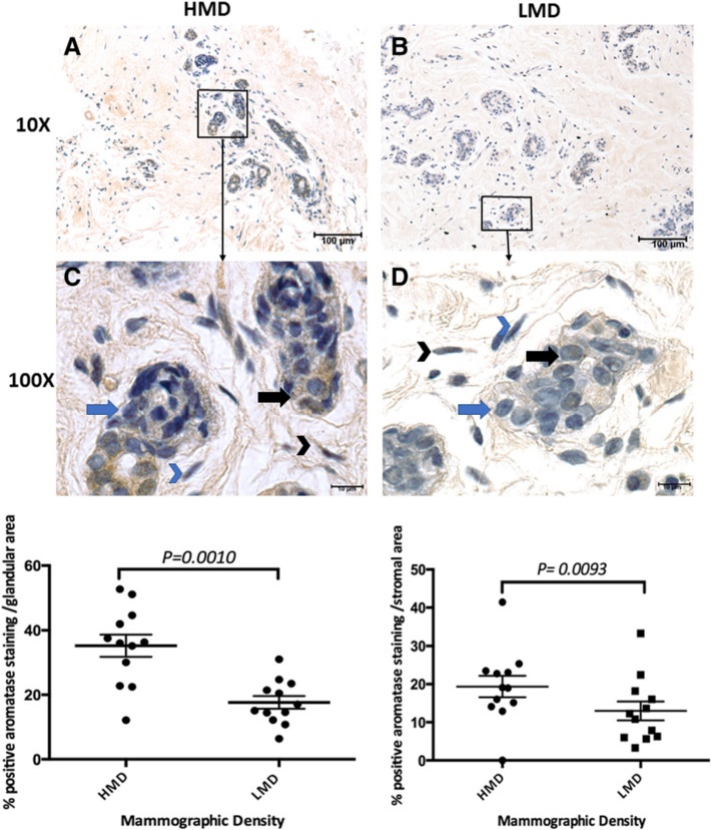
Aromatase analysis in high mammographic density (HMD) and low mammographic density (LMD) tissue samples. Cytoplasmic aromatase immunoreactivity appeared punctate in both epithelial and stromal components of paired HMD and LMD specimens (n = 15 women) (**a-d**). Percentages of positive aromatase expression were calculated by manually counting the positively stained cells (brown cytoplasm) and total number of cells in glandular areas and stroma, respectively (**e**). *Black arrows*: positive epithelial cell staining; *blue arrows*: negative epithelial staining; *black arrowheads*: positive stromal cell staining; *blue arrowheads*: negative stromal cell staining

Cells with positive cytoplasmic aromatase staining were counted. The percentages of aromatase-positive epithelial and stromal cells were significantly increased in HMD compared with LMD tissue (Fig. 5). Qualitatively, aromatase staining was observed to be more prevalent in the luminal compared with basal epithelial layer of HMD tissues (Fig. 5a, c).

### Macrophage staining

**Fig. 6:**
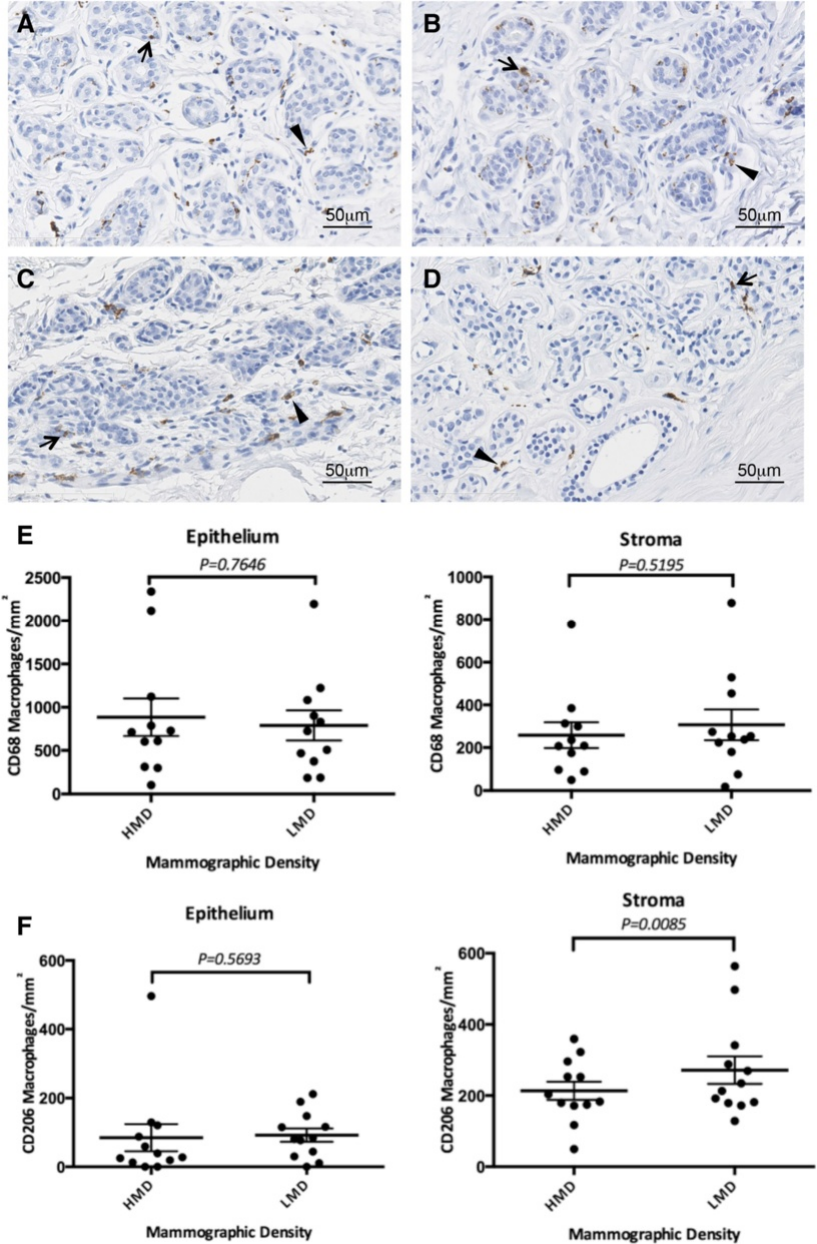
Macrophage staining. Macrophage abundance in low mammographic density (LMD) (**a**, **c**) and high mammographic density (HMD) (**b**, **d**) paired samples (n = 15) stained with pan-macrophage marker CD68 (A, B) and the C-type lectin receptor macrophage mannose receptor 1 (**c**, **d**). Epithelial cell–associated (*arrows*) and stromal cell–associated (*arrowheads*) macrophages were quantified per square millimetre of tissue in three randomly selected glandular and stromal areas within each tissue sample, and abundance was compared between LMD and HMD paired samples using the nonparametric Wilcoxon test for epithelium and the paired *t* test for stroma (data passed the D’Agostino-Pearson omnibus normality test) (*P < 0.05). Percentage changes in abundance of epithelial and stromal cell–associated CD68 (**e**) and CD206 (**f**) in HMD tissue compared with the LMD paired sample are shown

We assessed the paired HMD and LMD tissue sections for macrophage abundance, location and phenotype using CD68 and CD206 immunohistochemistry, as illustrated in Fig. 6. The overall number of macrophages (CD68^+^) was not significantly altered in either the epithelial or stromal compartments. However, within the HMD stromal regions, there was a significant decrease in the number of CD206^+^ alternatively activated macrophages (*P* < 0.01).

### Triple immunofluorescence staining for epithelial maturation and epithelial–mesenchymal transition

**Fig. 7:**
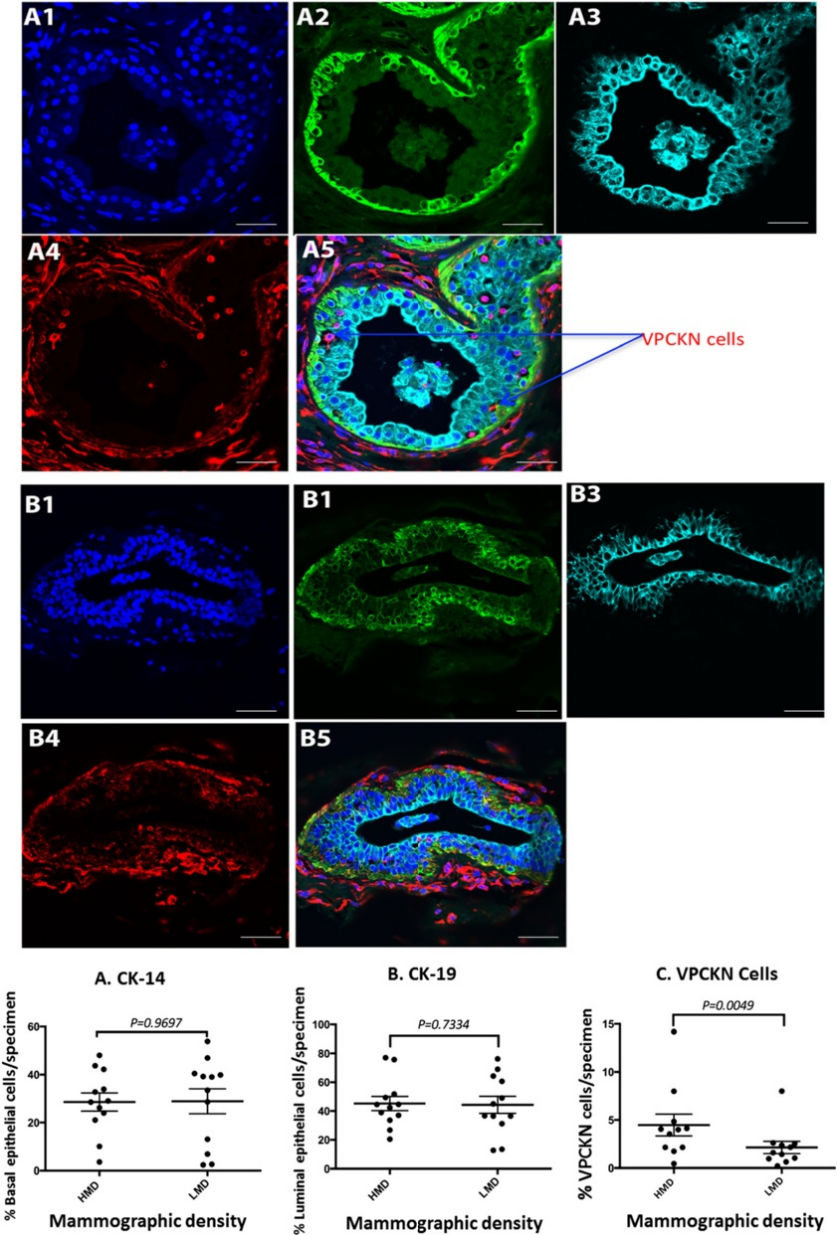
Cytokeratin (CK)-19, CK-14 and vimentin triple immunofluorescence staining. High mammographic density (HMD) (**a**) and low mammographic density (LMD) (**b**) tissue samples were stained with 4′,6-diamidino-2-phenylindole (**a** *1* and **b** *1*), CK-14 (**a** *2* and **b** *2*) CK-19 (**a** *3* and **b** *3*) and vimentin (**a** *4* and **b** *4*) and assessed as composite images (**a** *5* and **b** *5*). *Arrows* indicate cells inside the epithelial layer that stained negative for CK-19 or CK-14, but positive for vimentin. (**c**) Using the point-counting method, the percentages of CK-14 basal epithelial cells and CK-19 luminal epithelial cells were determined, as were the number of vimentin-expressing CK^−^ cells per whole glandular area of the tissue sections. The results for HMD and LMD paired samples were analysed using the nonparametric Wilcoxon matched-pairs signed-rank test. *VPCKN* vimentin-positive, cytokeratin-negative. Scale bar = 10 μm

**Fig. 8:**
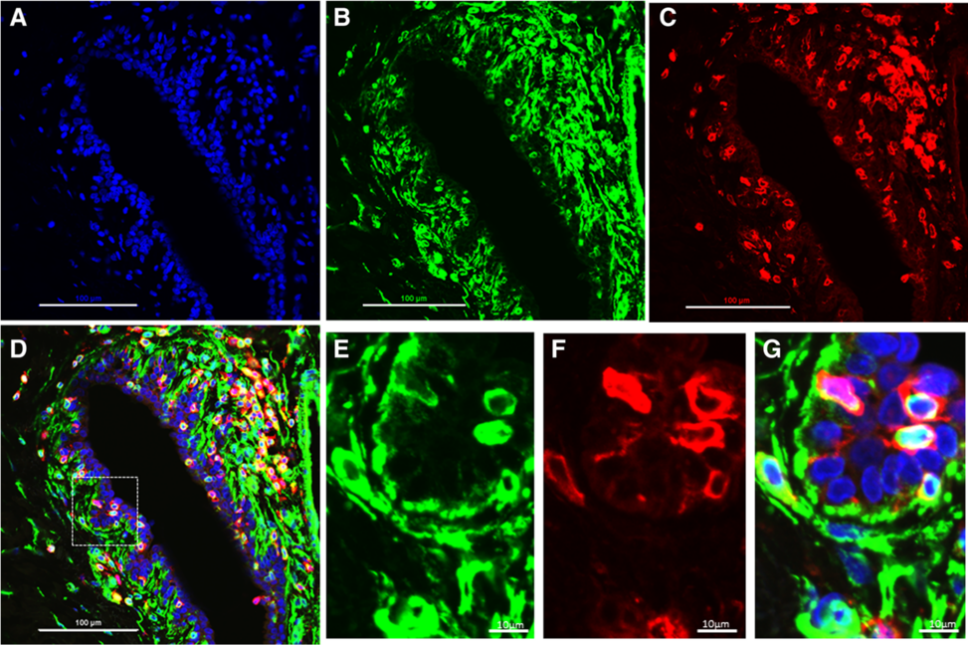
The vimentin^+^ cells in the epithelial layer are immune cells (CD45^+^). Immunofluorescence staining of high mammographic density (HMD) samples with 4′,6-diamidino-2-phenylindole (**a**), vimentin (**b**) and pan-CD45 immune cell marker (**c**) are shown as well as the overlay of all stain (**d**). (**e**-**g**) High-power images of the selected region in (D)

There was no significant difference in the proportions of basal (CK-14^+^) or luminal (CK-19^+^) epithelial cells between HMD and LMD specimens (Fig. 7). No vimentin^+^ and CK^+^ double-positive cells were seen in either HMD or LMD regions, ruling out any overt involvement of EMT. However, we observed a small minority of cells within the epithelium (not limited to the basal layer) that stained positive for vimentin and were negative for both CK-19 and CK-14 (Fig. 7). Using the same point-counting method, we found that the percentage of intraepithelial, vimentin-positive, cytokeratin-negative (VPCKN) cells was significantly increased in the HMD group compared with LMD. We further stained the specimens for pan-CK, CD68 and vimentin to determine if these VPCKN cells were macrophages. Whilst CD68^+^ cells were abundant in the epithelium (as well as the stroma), only 25 % to 50 % of the VPCKN cells were CD68^+^ (data not shown). To assess if they were immune cells, CD45 and vimentin double staining was used. All of the VPCKN cells within the epithelial regions were immune cells, as they costained with the pan-immune cell marker CD45 (Fig. 8). As expected, immune cells were also present throughout the stromal regions.

## Discussion

### Stromal and epithelial compartments increased in high mammographic density tissue

Adjusted for age and BMI, MD has consistently been shown to be a strong and independent risk factor for BC [31, 32]. Independent of its potential masking effect on tumours in a mammogram [1, 7, 33, 34], DCIS lesions were found to occur preferentially in HMD areas in a retrospective study where mammograms at BC diagnosis were compared with the most recent previous mammograms [35]. BCs that arise within HMD areas have also been shown in many studies to consist of features that suggest poor prognosis, such as high histological grade and lymphovascular invasion, compared with tumours that arise from LMD areas [27, 36–40].

The biological basis of MD has been investigated by various studies in the past decade, most of which were focused on malignant breast tissues [41–43]. In a cohort of 236 women who had normal breast tissue, Li and colleagues, in their autopsy study of accidental deaths, found significantly increased amounts of epithelial tissue in HMD breast tissue [44]. Hawes et al. examined the histology of 12 healthy premenopausal women and observed increased numbers of epithelial cells were associated with HMD, but they did not find any difference in proliferation using Ki-67 staining [13]. This result is consistent with other published data regarding cohorts of 66 women [45] and 59 women [11] of mixed menopausal status. However, we previously examined breast tissue in HMD and LMD regions of the same breast collected using an image-guided technique, and we did not observe a significant difference in the amount of epithelium between the two MD groups [12]. In this follow-up study of an extended cohort of such paired samples, we observed a small but significant increase in epithelial content of HMD compared with LMD regions in 41 women.

Whereas increased MD may be a partial consequence of increased epithelium, there was no elevated level of cellular proliferation, as demonstrated by the Ki-67 staining. In a small case–control study on postmenopausal women who received hormone therapy (HT) (28 receiving HT vs. 28 not receiving HT at the time of BC diagnosis), researchers found that HMD correlated with increased Ki-67 expression in ducts and lobules for both groups, although their data appear to be the only published data supporting a positive relationship between HMD and Ki-67, a result that may be mediated by the use of hormone therapy in those postmenopausal patients [46]. By contrast, Ghosh et al. did not observe a difference in Ki-67 expression when they analysed the core biopsies of dense and nondense regions of the same breast in a cohort of 59 healthy women who were either pre- or postmenopausal [11], which is in keeping with our findings in such a mixed cohort. In addition, among 344 women at high risk of developing BC, MD did not show any correlation with Ki-67 activity in a study in which investigators used tissues obtained by random periareolar fine-needle aspiration [47]. The assessment of cell proliferation can be challenging, as it is influenced by many factors, such as the menstrual cycle, hormone therapy and parity status. A strength of our study lies in the paired within-individual assessment, which eliminated these potential confounders. In regard to MD-associated studies on cancerous mammary tissue, a large case-only study involving 1975 patients with BC found no significant difference in the percentage MD between women who had high or low Ki-67 values in their BCs [48]. Also, in a case–control study of 279 patients with BC and 159 controls, MD was not associated with significantly increased proliferation markers in breast tissue [49]. The null association between MD and Ki-67 suggests that HMD may increase BC risk through a mechanism that is separate from increased cellular proliferation.

Increased stroma is the leading contributing factor for HMD; thus, it is not surprising that there have been studies showing that changes in the stromal composition alone lad to epithelial cancers [20, 50–52]. The breast stroma is composed of extracellular matrix (ECM), which not only provides an ultrastructure to support cellular growth but also contributes to cell adhesion, migration, differentiation and maintenance [19, 53–56]. In addition, ECM was shown to participate in tumorigenesis and progression of BC through cell surface receptors such as integrins and growth factors [57–59].

We recently suggested [32] that further analysis of the collagen content and orientation may shed light on HMD-associated BC risk, as studies of collagen alignment in rats showed that parous rats, which have reduced BC incidence, had less linearized collagen than nulliparous rats [60]. The MTB staining showed that the increased stromal area (and concurrently decreased adipose tissue) was also associated with an increase in collagen-rich tissue. The densely packed collagens in HMD tissue play a key role in stromal stiffness*.* This possibility has been supported by various investigations of stromal ECM, proteoglycan expression and collagen content in mammary tissue [17, 18, 20, 52, 61–63]. In those studies, researchers found that HMD was characterised by greater ECM stiffness, increased expression of lumican and decorin and increased amounts of collagen. In addition, Conklin and colleagues found that tumour-associated collagen signature-3 (TACS-3), a collagen alignment marker, was positively associated with expression of stromal syndecan 1, a receptor for several ECM proteins, including collagen. Furthermore, TACS-3 also correlated with poor disease-free survival in 196 patients, suggesting that, in addition to collagen content, the architecture and collagen alignment may be key in tumorigenesis and invasiveness [64]. We assessed the collagen organisation using GLCM analysis of SHG images and found that HMD tissue samples have a higher organisation than LMD tissue samples. This is consistent with the work of Provenzano et al. showing that breast tumour cells often localize near dense collagen or promote a desmoplastic response [20]. Using Wnt-1 tumour-bearing mice, they showed the presence of increased collagen in areas of hyperplasia before the development of palpable tumours [41].

Fat as a breast tissue fraction often is not considered to play an active role in MD-associated BC risk. However, Lokate and colleagues found, in a nested case–control study (358 cases and 859 controls; postmenopausal women), that local breast fat increased BC risk independently of BMI. They also noted that the relationship with BC is stronger for HMD than for LMD areas, with the final risk being dependent on the breast composition [65]. Pettersson et al., in a larger case–control study [464 cases and 998 controls (premenopausal women) and 960 cases and 1662 controls (postmenopausal women)], found that nondense breast area was associated with decreased BC risk among both groups of women, and they proposed that local adipose tissue in the breast may have a protective role against BC [66]. Adipose tissue is known to contain active endocrine cells that may contribute to perturbations in the breast microenvironment [67, 68]. For example, oestrogen produced by fat through conversion of androgens by aromatase has been shown to increase BC risk [69–71]. Adipokines, such as leptin, which are secreted by fat tissue, also contribute to the development of BC by stimulating oestrogen biosynthesis and BC cell growth [72–74]. Although increased adipose tissue overall in the form of a higher BMI may lead to raised circulating and/or local levels of oestrogen and subsequent BC risk, fatty breast tissue per se may reduce BC risk by reducing the chance of promalignancy interactions between epithelial cells and the stroma.

The difference in histological composition assessed by using an MD tissue index correlated with results that were analysed using JMicroVision for our 41 paired specimens (see Additional file 1). Hence, where mammograms or X-rays are not available, an MD tissue index could be a valuable substitute tool to guide the selection of HMD and LMD breast tissue for future studies.

### High mammographic density tissue has increased aromatase but no changes in hormone receptor expression

With regard to hormonal modulation of MD, increased MD can be observed during the luteal phase of the menstrual cycle, correlating with a woman’s increased serum levels of circulating endogenous oestradiol and progesterone. MD then decreases during the menstrual phase and plateaus during the follicular phase, despite high oestradiol levels in that period. Thus, it is likely that oestradiol and progesterone synergistically cause a transient rise in MD in the luteal phase [4]. We showed that MD of human breast tissue in our xenograft murine engineering chambers was influenced by the murine peripartum states as well as by tamoxifen treatment of the nonobese diabetic/severe combined immunodeficiency NOD SCID mice [75, 76]. Our unique within-individual paired analyses eliminated confounders such as different stages of the menstrual cycle or menopausal status among women; however, we did not observe any significant difference in the percentage expression of ER and PR between HMD and LMD tissue within the same individual. This is consistent with Ghosh and colleagues’ finding that the relative expression of ER and PR was similar in dense and nondense tissue of 24 women [11]. Data derived from published epidemiological studies also showed that, although MD was more strongly associated with ER-positive than ER-negative BC for women under the age of 55, HMD is strongly associated with all BC subtypes [77, 78]. Although in the majority of previous studies researchers have reported a null association between MD and ER or PR status, Ding et al. found that MD was a stronger risk factor for ER-positive BC than for ER-negative BC in a group of 370 BC cases with 1904 age-matched controls [36]. Overall, this area remains a controversial topic, and the mechanisms behind these conflicting findings are poorly understood.

Aromatase is the key driver for local oestrogen production within the breast after menopause. It is expressed by the adipose stromal cells and encoded by the *CYP19A1* gene [79, 80]. Aromatase transfection stimulates BC cell growth in vitro, perhaps via increased production of oestrogen [81], and increased aromatase mRNA levels were found in BC compared with normal breast tissue [82]. Treatment of ER^+^ BC in postmenopausal women with aromatase inhibitors (AIs) has demonstrated the important role that aromatase plays in the extraovarian oestrogen biosynthesis, and it is the treatment of choice for postmenopausal ER^+^ tumours [83]. Forced expression of aromatase in mouse mammary glands led to hyperplasia and tumour growth, suggesting aromatase’s tumorigenic potential when its expression becomes aberrant [84]. In a recent study on the breast fat precursor cells (referred to as *human adipose stromal cells*) isolated from women who underwent reduction mammoplasty, researchers found that high cell culture density and increased ECM presence significantly induced aromatase expression in vitro [85]. Additionally, Vachon and coworkers found, in a group of 49 healthy women aged 40–82 years, that aromatase immunoreactivity was significantly higher in epithelial and stromal cells of dense regions than in nondense tissues sampled using core biopsies [86]. In keeping with their findings, we also observed significantly increased levels of aromatase expression in both epithelial and stromal components of HMD tissue compared with LMD. Increased aromatase expression in HMD regions (increased stroma and epithelium, but less fat) may be reflective of a higher number of immature stromal preadipocytes, which is a source of aromatase activity [87, 88].

In the large International Breast Cancer Intervention Study (IBIS) I prevention trial, investigators assessed the effect of tamoxifen on MD in healthy women and demonstrated a significant reduction of MD after 12–18 months of therapy [89]. This finding reflected MD as a hormonally modulated phenotype, despite its lack of association with ER^+^ or ER^−^ BCs. On the basis of our results, aromatase expression, but not ER and/or PR status, appears to correlate with HMD. To date, researchers in several small studies (less than 100 women with study period ranging from 6 to 24 months) have published results of changes in MD in response to AIs. These results are largely conflicting and affected by factors such as low baseline MD and patients previously receiving long-term tamoxifen before commencement of AIs [47, 90, 91]. More recently, Henry et al. observed a significant decrease in mean percentage MD (from 17.1 % to 15.1 %) in 259 postmenopausal women with BC who were initiating adjuvant AI therapy and randomly assigned to treatment with exemestane (25 mg) or letrozole (2.5 mg) daily for 2 years [92]. In a case–control study where 387 patients with postmenopausal BC were treated with either anastrozole (1 mg daily) or exemestane (25 mg daily), 10-month AI therapy was associated with a 5 % or greater decrease in MD in 56 cases. However, this reduction was not significant compared with their controls, which were matched for age, BMI and baseline MD [42]. In the IBIS-II trial, investigators have also found a 53 % risk reduction in women aged 40 to 70 years who took 1 mg of oral anastrozole for 5 years (1920 cases and 1944 controls) for BC prevention [93]. However, MD has not yet been assessed in association with the risk reduction.

### High mammographic density tissue has lower abundance of CD206^+^ alternatively activated macrophages

Macrophages in established tumours exert well-described effects on angiogenesis, invasion, metastasis and suppression of antitumour immunity [94, 95]. However, the relationship between macrophages and tumorigenesis is complex, as macrophages are also involved in immune surveillance that protects against tumorigenesis, as well as in tumour-initiating roles such as production of DNA-damaging agents, including reactive oxygen species and reactive nitrogen intermediates. Macrophage functions can be classified on the basis of how they are activated. Classically activated (M1) macrophages are activated by interferon γ (IFNγ) and microbial products, whereas alternatively activated (M2) macrophages are activated by interleukin (IL)-4, IL-10 and IL-13. M1 macrophages express high levels of proinflammatory cytokines, are able to kill pathogens and can prime antitumour responses, whereas M2 macrophages exhibit increased expression of IL-10 anti-inflammatory cytokines. The majority of tumour-associated macrophages are alternatively activated, as they promote tumour angiogenesis and tissue remodelling and their major product, IL-10, has known immunosuppressive roles [96, 97].

We found that the abundance of alternatively activated CD206^+^ macrophages was significantly reduced in HMD tissue compared with LMD tissue. Although the role of macrophages in tumour initiation and the early stages of tumorigenesis in BC is largely unknown, it is plausible that in normal breast tissue the alternatively activated macrophages assist in dampening immune responses, thus reducing the risk of inflammation-driven tumour initiation [98]. Interestingly, macrophages have been identified as key cells in the fibrillogenesis of collagen during mammary gland development in mice [62], and they may play a similar role in promoting increased organization of collagen associated with HMD. Furthermore, Morris et al. observed elevated aromatase expression as well as macrophages forming crown-like structures (CLSs) surrounding necrotic adipocytes in the mammary glands of obese mice as well as in tissue samples of 14 obese women [99] using a CD68 antibody that also stains for other myeloid cells and fibroblasts [100]. They postulated that the CLSs could be a useful biomarker for increased BC risk. We did not observe CLSs in the HMD and LMD samples after staining them with a CD68 antibody that we have found to be specific for macrophages. This could also be due to the fact our cohort was not specifically selected to include obese women.

### Similar abundance of basal and luminal cells, but increased vimentin^+^ immune cells in the epithelial layer of high mammographic density tissue

We were able to demonstrate increased epithelial compartments in HMD compared with LMD in our study, but we were also interested in determining whether the increased MD correlated with a more immature epithelial cell compartment. We assessed the proportion of basal and luminal cells within the HMD tissue using CK-14, CK-19 and vimentin triple IF staining and showed that it was not significantly altered compared with LMD. Vimentin was also included as a test for evidence of EMT, which has been implicated in many aspects of cancer progression and cancer initiation [22, 101]. In particular, Raviraj and colleagues observed EMT in solitary primary tumour cells in dense collagen stroma, as these cells were positive for both vimentin and CK-19 [102]. However, no such overt evidence of EMT was seen in either LMD or HMD specimens in our study. Vimentin staining was observed surrounding the glandular tissue, where it stained both stromal fibroblasts and some of the basal myoepithelial cells, which also costained with CK-14.

To our surprise, we noticed that there were aberrantly positioned vimentin^+^ cells within the luminal compartment of the epithelial cell layer that were negative for CK-14 and CK-19, suggesting that they were not myoepithelial cells or mature epithelial cells undergoing EMT. To probe the identity of these cells, we costained with vimentin and a pan-immune cell marker, CD45. The vimentin^+^ cells in the epithelial layer that were not CK^+^ were positively identified as CD45^+^ immune cells. Further analysis demonstrated that a subset of these cells were also CD68^+^ macrophages (data not shown). This suggests that the HMD samples have increased vimentin^+^ immune cells present within the epithelial layer, which may lead to increased local inflammation.

Inflammation in cancer development bears the analogy of a double-edged sword: on one hand, lymphocytes actively seek out and eradicate dysplastic cells to suppress tumour formation; on the other hand, cancers have been observed to arise from areas of chronic inflammation [32, 103]. The important role of immunosurveillance in cancer prevention has been demonstrated in immunodeficient mice such as IFNγ-deficient mice [104]. In humans, patients with innately deficient immune systems or those who with immunosuppression due to medical therapy, have an increased risk of developing certain types of cancer, such as Hodgkin’s lymphoma and Kaposi sarcoma [105]. Interestingly, where local inflammation remains chronic, such as in cases of inflammatory bowel disease or chronic *Helicobacter pylori* infection of the stomach, the frequency of cancer is also increased [106]. Traditionally, BCs are not viewed to be immunogenic, as the incidence is not increased in patients with immunodeficiency [107]. However, Hussein and colleagues [108] found that CD3^+^ T cells and CD20^+^ B cells were significantly increased in cancerous breast tissue compared with benign breast tissue in 53 mastectomy cases. Few studies have attempted to measure whether the effect of inflammation on breast carcinogenesis is mediated through independent effects on MD. In a case–control study of 542 postmenopausal women, Reeves et al*.* did not find any association of IL-6, tumour necrosis factor-α or C-reactive protein with MD upon adjustment for BMI [109]. Also, the Australian Mammographic Density Twins and Sisters Study researchers reported no association between MD with the use of aspirin and other nonsteroidal anti-inflammatory drug (NSAID) use in 3286 women, based on multiple linear regression analysis [110]. Nevertheless, using paired analysis of HMD and LMD samples, we observed that the epithelium in HMD exhibited increased numbers of vimentin^+^ immune cell infiltration, whereas the stromal area in HMD had reduced numbers of alternatively activated macrophages, suggesting an altered balance between anti- and proinflammatory signals in HMD epithelium and stroma. Only a proportion of vimentin^+^/CD45^+^ cells stained positive for macrophages using the pan-macrophage marker CD68. CD45 is a pan-inflammatory marker that stains positive for most cells of hematopoietic origin, such as adaptive immune (T and B lymphocytes) cells, as well as myeloid-derived suppressor cells. Our data suggest that the abundance and phenotype of immune system cells are altered in the microenvironment in HMD tissue. Although we specifically investigated macrophages, it is likely that other components are also affected by MD. Further analysis of the identity of the CD45^+^/vimentin^+^ population that we have shown, as well as comparison of T and B cell populations surrounding lobules and ducts in HMD and LMD samples, may reveal further differences in the immune microenvironment that affects BC risk, as has been described previously [111].

### Strengths and limitations

We acknowledge that MD of the breast is greatly influenced by age, BMI, menopausal status and menstrual cycle, and immune cell infiltration along with collagen level could also vary with parity status [60, 112]. Although our within-individual matched-pairs design eliminated such confounders from our comparison of HMD and LMD tissue specimens from the same woman, we may still see differences in the size and/or scope of HMD/LMD differential effects, depending on parity and parity-related issues. The sampling of non-neoplastic breast tissue enabled us to examine features unique to HMD specimens that may be responsible for increased BC risk before tumorigenesis takes place. Our study cohort, however, was a group of predominantly premenopausal and parous women with an elevated BC risk compared with the average population and thus may not be fully representative of the general population. However, MD was not shown to bear any relationship with *BRCA1/2* carrier status versus *BRCA1/2*^−^ but familial high-risk women (approximately one-third of the cases are not *BRCA1/2* carriers) [12, 25]. *BRCA1/2* status does not affect MD [113], and MD confers an elevated risk in *BRCA1/2* carriers similar to that of sporadic women [114]. Therefore, it is likely that the compositional and cellular changes we observed in this study population are translatable to the general population.

## Conclusions

This study confirms that HMD breast tissue is composed of significantly greater proportions of epithelium, stroma and collagen, and less adipose tissue, than LMD regions of the same breast. This finding has been reported in previously published studies. Consistent with most published data, our findings were that there were similar levels of ER, PR and Ki-67 expression, as well as increased aromatase immunoreactivity, in HMD tissue. To date, there is no evidence in the literature on the relationship of MD with collagen architecture and orientation, macrophage infiltration, subepithelial cell proportions or the level of immune cell influx. To our knowledge, the present study is the first to show that HMD tissue has enhanced stromal collagen organisation, significantly reduced stromal alternatively activated macrophage infiltration and increased abundance of vimentin^+^ immune cells within the epithelial compartment. Increased BC risk associated with high MD is likely a result of longitudinally cumulative exposure to increased epithelium, dense stromal architecture and various growth and hormonal factors in a breast microenvironment that promotes mitosis, inflammation and subsequent malignant transformation.

## Additional files

Additional file 1:
**MD semiquantitative assessment using the MD tissue index.** Upon reviewing H&E-stained slides, tissue samples were assigned to one of the five categories: (1) 0 % to 10 % of tissue was stroma and/or epithelium/tissue section (**A**); (2) 11 % to 25 % of tissue was stroma and/or epithelium/tissue section (**B**); (3) 26 % to 50 % of tissue was stroma and/or epithelium/tissue section (**C**); (4) 51 % to 75 % of tissue was stroma and/or epithelium/tissue section (**D**); and (5) >75 % of tissue was stroma and/or epithelium/tissue section (**E**). (**F**) Scatterplot of assigned density category based on the percentage of stroma and epithelium per specimen shows that this categorical method of assessing MD produced results consistent with those derived from JMicroVision analyses as shown in Fig. 3 (n = 41 women). *HMD* high mammographic density, *LMD* low mammographic density. Owing to the different sizes of whole tissue sections, we show entire tissues, hence the inconsistencies of scales shown. Additional file 2:
**Assessment of histological composition for parous and premenopausal women.** (**A**) The distribution of participants by their parity and menopausal status. (**B**) The quantitative analyses by Wilcoxon matched-pairs signed-rank test of stroma, epithelium and fat percentages in premenopausal women [subset a (n = 37) shown in (A)]. (**C**) Quantitative analyses of stroma, epithelium and fat percentages in premenopausal women [subset b (n = 28) in (A)]. (**D**) Quantitative analyses of stroma, epithelium and fat percentages in parous and premenopausal women [subset d (n = 25) in (A)]. *HMD* high mammographic density, *LMD* low mammographic density. Additional file 3:
**Assessment of histological composition for nulliparous and peri- or postmenopausal women.** Quantitative analyses by Wilcoxon matched-pairs signed rank test of the histological compositions of nulliparous women (n = 4) (**A**), perimenopausal women (n = 5) (**B**), postmenopausal women (n = 8) (**C**) and peri- and postmenopausal women (n = 13) (**D**). *HMD* high mammographic density, *LMD* low mammographic density.

## Abbreviations

AI: Aromatase inhibitor
BC: Breast cancer
BI-RADS: Breast Imaging-Reporting and Data System
BMI: Body mass index
CK: Cytokeratin
CLS: Crown-like structure
DCIS: Ductal carcinoma in situ
ECM: Extracellular matrix
EMT: Epithelial–mesenchymal transition
ER: Oestrogen receptor
GLCM: Grey-level co-occurrence matrix
H&E: Haematoxylin and eosin
HMD: High mammographic density
HT: Hormone therapy
IBIS: International Breast Cancer Intervention Study
IF: Immunofluorescence
IFNγ: Interferon γ
IL: Interleukin
LMD: Low mammographic density
M1: Classically activated macrophage
M2: Alternatively activated macrophage
MD: Mammographic density
MTB: Masson’s trichrome blue
NSAID: Nonsteroidal anti-inflammatory drug
PBS: Phosphate-buffered saline
PR: Progesterone receptor
ROI: Region of interest
SHG: Second harmonic generation
TACS-3: Tumour-associated collagen signature-3
VPCKN: Vimentin-positive, cytokeratin-negative

## References

[1] McCormack VA, dos Santos SI (2006). Breast density and parenchymal patterns as markers of breast cancer risk: a meta-analysis. Cancer Epidemiol Biomarkers Prev.

[2] Wolfe JN (1976). Breast patterns as an index of risk for developing breast cancer. AJR Am J Roentgenol.

[3] Boyd NF, Byng JW, Jong RA, Fishell EK, Little LE, Miller AB (1995). Quantitative classification of mammographic densities and breast cancer risk: results from the Canadian National Breast Screening Study. J Natl Cancer Inst.

[4] Stomper PC, D’Souza DJ, DiNitto PA, Arredondo MA (1996). Analysis of parenchymal density on mammograms in 1353 women 25–79 years old. AJR Am J Roentgenol.

[5] Newman B, Mu H, Butler LM, Millikan RC, Moorman PG, King MC (1998). Frequency of breast cancer attributable to *BRCA1* in a population-based series of American women. JAMA.

[6] Peto J, Collins N, Barfoot R, Seal S, Warren W, Rahman N (1999). Prevalence of BRCA1 and BRCA2 gene mutations in patients with early-onset breast cancer. J Natl Cancer Inst.

[7] Boyd NF, Guo H, Martin LJ, Sun L, Stone J, Fishell E (2007). Mammographic density and the risk and detection of breast cancer. N Engl J Med.

[8] Sprague BL, Gangnon RE, Burt V, Trentham-Dietz A, Hampton JM, Wellman RD, et al. Prevalence of mammographically dense breasts in the United States. J Natl Cancer Inst. 2014;106:dju255. doi:10.1093/jnci/dju255. 10.1093/jnci/dju255 PMC4200066 25217577

[9] *Are You Dense?* http://www.areyoudense.org/. Accessed 4 Jun 2015.

[10] Rhodes DJ, Conners AL (2014). Breast density legislation: implications for patients and primary care providers. Minn Med.

[11] Ghosh K, Brandt KR, Reynolds C, Scott CG, Pankratz VS, Riehle DL (2012). Tissue composition of mammographically dense and non-dense breast tissue. Breast Cancer Res Treat.

[12] Lin SJ, Cawson J, Hill P, Haviv I, Jenkins M, Hopper JL (2011). Image-guided sampling reveals increased stroma and lower glandular complexity in mammographically dense breast tissue. Breast Cancer Res Treat.

[13] Hawes D, Downey S, Pearce CL, Bartow S, Wan P, Pike MC (2006). Dense breast stromal tissue shows greatly increased concentration of breast epithelium but no increase in its proliferative activity. Breast Cancer Res.

[14] Baschieri F, Confalonieri S, Bertalot G, Di Fiore PP, Dietmaier W, Leist M (2014). Spatial control of Cdc42 signalling by a GM130–RasGRF complex regulates polarity and tumorigenesis. Nat Commun.

[15] Chen N, Balasenthil S, Reuther J, Killary AM (2014). DEAR1, a novel tumor suppressor that regulates cell polarity and epithelial plasticity. Cancer Res.

[16] Arendt LM, Rudnick JA, Keller PJ, Kuperwasser C (2010). Stroma in breast development and disease. Semin Cell Dev Biol.

[17] Alowami S, Troup S, Al-Haddad S, Kirkpatrick I, Watson PH (2003). Mammographic density is related to stroma and stromal proteoglycan expression. Breast Cancer Res.

[18] Barcus CE, Keely PJ, Eliceiri KW, Schuler LA (2013). Stiff collagen matrices increase tumorigenic prolactin signaling in breast cancer cells. J Biol Chem.

[19] Hay ED (1993). Extracellular matrix alters epithelial differentiation. Curr Opin Cell Biol.

[20] Provenzano PP, Inman DR, Eliceiri KW, Knittel JG, Yan L, Rueden CT (2008). Collagen density promotes mammary tumor initiation and progression. BMC Med.

[21] Grivennikov SI, Greten FR, Karin M (2010). Immunity, inflammation, and cancer. Cell.

[22] Thiery JP, Acloque H, Huang RY, Nieto MA (2009). Epithelial-mesenchymal transitions in development and disease. Cell.

[23] van Denderen BJ, Thompson EW (2013). Cancer: the to and fro of tumour spread. Nature.

[24] National Health and Medical Research Council, Australian Research Council, Australian Vice-Chancellors’ Committee. National Statement on Ethical Conduct in Human Research. Canberra: Australian Government; 2007. http://www.nhmrc.gov.au/_files_nhmrc/file/publications/synopses/e72.pdf. Accessed 4 Jun 2015.

[25] Chew GL, Huang D, Lin SJ, Huo C, Blick T, Henderson MA (2012). High and low mammographic density human breast tissues maintain histological differential in murine tissue engineering chambers. Breast Cancer Res Treat.

[26] Ham S, Meachem SJ, Choong CS, Charles AK, Baynam GS, Jones TW (2013). Overexpression of aromatase associated with loss of heterozygosity of the *STK11* gene accounts for prepubertal gynecomastia in boys with Peutz-Jeghers syndrome. J Clin Endocrinol Metab.

[27] Aiello EJ, Buist DS, White E, Porter PL (2005). Association between mammographic breast density and breast cancer tumor characteristics. Cancer Epidemiol Biomarkers Prev.

[28] Cicchi R, Kapsokalyvas D, De Giorgi V, Maio V, Van Wiechen A, Massi D (2010). Scoring of collagen organization in healthy and diseased human dermis by multiphoton microscopy. J Biophotonics.

[29] Nobis M, McGhee EJ, Morton JP, Schwarz JP, Karim SA, Quinn J (2013). Intravital FLIM-FRET imaging reveals dasatinib-induced spatial control of Src in pancreatic cancer. Cancer Res.

[30] Norwegian University for Life Sciences NMBU ImageJ Plugins. http://arken.umb.no/~kkvaal/eamtexplorer/imagej_plugins.html. Accessed 4 Jun 2015.

[31] Huo CW, Chew GL, Britt KL, Ingman WV, Henderson MA, Hopper JL (2014). Mammographic density-a review on the current understanding of its association with breast cancer. Breast Cancer Res Treat.

[32] Britt K, Ingman W, Huo C, Chew G, Thompson E (2014). The pathobiology of mammographic density. J Cancer Biol Res.

[33] Porter GJ, Evans AJ, Cornford EJ, Burrell HC, James JJ, Lee AH (2007). Influence of mammographic parenchymal pattern in screening-detected and interval invasive breast cancers on pathologic features, mammographic features, and patient survival. AJR Am J Roentgenol.

[34] Mandelson MT, Oestreicher N, Porter PL, White D, Finder CA, Taplin SH (2000). Breast density as a predictor of mammographic detection: comparison of interval- and screen-detected cancers. J Natl Cancer Inst.

[35] Ursin G, Hovanessian-Larsen L, Parisky YR, Pike MC, Wu AH (2005). Greatly increased occurrence of breast cancers in areas of mammographically dense tissue. Breast Cancer Res.

[36] Ding J, Warren R, Girling A, Thompson D, Easton D (2010). Mammographic density, estrogen receptor status and other breast cancer tumor characteristics. Breast J.

[37] Sala E, Solomon L, Warren R, McCann J, Duffy S, Luben R (2000). Size, node status and grade of breast tumours: association with mammographic parenchymal patterns. Eur Radiol.

[38] Fasching PA, Heusinger K, Loehberg CR, Wenkel E, Lux MP, Schrauder M (2006). Influence of mammographic density on the diagnostic accuracy of tumor size assessment and association with breast cancer tumor characteristics. Eur J Radiol.

[39] Kerlikowske K, Cook AJ, Buist DS, Cummings SR, Vachon C, Vacek P (2010). Breast cancer risk by breast density, menopause, and postmenopausal hormone therapy use. J Clin Oncol.

[40] Yaghjyan L, Colditz GA, Collins LC, Schnitt SJ, Rosner B, Vachon C (2011). Mammographic breast density and subsequent risk of breast cancer in postmenopausal women according to tumor characteristics. J Natl Cancer Inst.

[41] Bland KI, Kuhns JG, Buchanan JB, Dwyer PA, Heuser LF, O’Connor CA (1982). A clinicopathologic correlation of mammographic parenchymal patterns and associated risk factors for human mammary carcinoma. Ann Surg.

[42] Bright RA, Morrison AS, Brisson J, Burstein NA, Sadowsky NS, Kopans DB (1988). Relationship between mammographic and histologic features of breast tissue in women with benign biopsies. Cancer.

[43] Fisher ER, Palekar A, Kim WS, Redmond C (1978). The histopathology of mammographic patterns. Am J Clin Pathol.

[44] Li T, Sun L, Miller N, Nicklee T, Woo J, Hulse-Smith L (2005). The association of measured breast tissue characteristics with mammographic density and other risk factors for breast cancer. Cancer Epidemiol Biomarkers Prev.

[45] Reeves KW, Stone RA, Modugno F, Ness RB, Vogel VG, Weissfeld JL (2009). Longitudinal association of anthropometry with mammographic breast density in the Study of Women’s Health Across the Nation. Int J Cancer.

[46] Harvey JA, Santen RJ, Petroni GR, Bovbjerg VE, Smolkin ME, Sheriff FS (2008). Histologic changes in the breast with menopausal hormone therapy use: correlation with breast density, estrogen receptor, progesterone receptor, and proliferation indices. Menopause.

[47] Khan QJ, Kimler BF, O’Dea AP, Zalles CM, Sharma P, Fabian CJ (2007). Mammographic density does not correlate with Ki-67 expression or cytomorphology in benign breast cells obtained by random periareolar fine needle aspiration from women at high risk for breast cancer. Breast Cancer Res.

[48] Heusinger K, Jud SM, Häberle L, Hack CC, Fasching PA, Meier-Meitinger M (2012). Association of mammographic density with the proliferation marker Ki-67 in a cohort of patients with invasive breast cancer. Breast Cancer Res Treat.

[49] Verheus M, Maskarinec G, Erber E, Steude JS, Killeen J, Hernandez BY (2009). Mammographic density and epithelial histopathologic markers. BMC Cancer.

[50] Li X, Placencio V, Iturregui JM, Uwamariya C, Sharif-Afshar AR, Koyama T (2008). Prostate tumor progression is mediated by a paracrine TGF-β/Wnt3a signaling axis. Oncogene.

[51] Trimboli AJ, Cantemir-Stone CZ, Li F, Wallace JA, Merchant A, Creasap N (2009). *Pten* in stromal fibroblasts suppresses mammary epithelial tumours. Nature.

[52] Provenzano PP, Inman DR, Eliceiri KW, Keely PJ (2009). Matrix density-induced mechanoregulation of breast cell phenotype, signaling and gene expression through a FAK–ERK linkage. Oncogene.

[53] Lin CQ, Bissell MJ (1993). Multi-faceted regulation of cell differentiation by extracellular matrix. FASEB J.

[54] Hojilla CV, Mohammed FF, Khokha R (2003). Matrix metalloproteinases and their tissue inhibitors direct cell fate during cancer development. Br J Cancer.

[55] Petersen OW, Rønnov-Jessen L, Howlett AR, Bissell MJ (1993). Interaction with basement membrane serves to rapidly distinguish growth and differentiation pattern of normal and malignant human breast epithelial cells. Proc Natl Acad Sci U S A. 1992;89(19):9064–8. A published erratum appears in. Proc Natl Acad Sci U S A.

[56] Li ML, Aggeler J, Farson DA, Hatier C, Hassell J, Bissell MJ (1987). Influence of a reconstituted basement membrane and its components on casein gene expression and secretion in mouse mammary epithelial cells. Proc Natl Acad Sci U S A.

[57] Juliano RL, Haskill S (1993). Signal transduction from the extracellular matrix. J Cell Biol.

[58] Taipale J, Keski-Oja J (1997). Growth factors in the extracellular matrix. FASEB J.

[59] Lochter A, Bissell MJ (1995). Involvement of extracellular matrix constituents in breast cancer. Semin Cancer Biol.

[60] Maller O, Hansen KC, Lyons TR, Acerbi I, Weaver VM, Prekeris R (2013). Collagen architecture in pregnancy-induced protection from breast cancer. J Cell Sci.

[61] Keely PJ (2011). Mechanisms by which the extracellular matrix and integrin signaling act to regulate the switch between tumor suppression and tumor promotion. J Mammary Gland Biol Neoplasia.

[62] Ingman WV, Wyckoff J, Gouon-Evans V, Condeelis J, Pollard JW (2006). Macrophages promote collagen fibrillogenesis around terminal end buds of the developing mammary gland. Dev Dyn.

[63] Guo YP, Martin LJ, Hanna W, Banerjee D, Miller N, Fishell E (2001). Growth factors and stromal matrix proteins associated with mammographic densities. Cancer Epidemiol Biomarkers Prev.

[64] Conklin MW, Eickhoff JC, Riching KM, Pehlke CA, Eliceiri KW, Provenzano PP (2011). Aligned collagen is a prognostic signature for survival in human breast carcinoma. Am J Pathol.

[65] Lokate M, Peeters PH, Peelen LM, Haars G, Veldhuis WB, van Gils CH (2011). Mammographic density and breast cancer risk: the role of the fat surrounding the fibroglandular tissue. Breast Cancer Res.

[66] Pettersson A, Hankinson SE, Willett WC, Lagiou P, Trichopoulos D, Tamimi RM (2011). Nondense mammographic area and risk of breast cancer. Breast Cancer Res.

[67] Chamras H, Bagga D, Elstner E, Setoodeh K, Koeffler HP, Heber D (1998). Preadipocytes stimulate breast cancer cell growth. Nutr Cancer.

[68] Roth J, Qiang X, Marbán SL, Redelt H, Lowell BC (2004). The obesity pandemic: where have we been and where are we going?. Obes Res.

[69] Szymczak J, Milewicz A, Thijssen JH, Blankenstein MA, Daroszewski J (1998). Concentration of sex steroids in adipose tissue after menopause. Steroids.

[70] Thijssen JH (2004). Local biosynthesis and metabolism of oestrogens in the human breast. Maturitas.

[71] Cleary MP, Grossmann ME (2009). Obesity and breast cancer: the estrogen connection. Endocrinology.

[72] Cleary MP, Grossmann ME, Ray A (2010). Effect of obesity on breast cancer development. Vet Pathol.

[73] Grossmann ME, Ray A, Nkhata KJ, Malakhov DA, Rogozina OP, Dogan S (2010). Obesity and breast cancer: status of leptin and adiponectin in pathological processes. Cancer Metastasis Rev.

[74] Brown KA, McInnes KJ, Hunger NI, Oakhill JS, Steinberg GR, Simpson ER (2009). Subcellular localization of cyclic AMP-responsive element binding protein-regulated transcription coactivator 2 provides a link between obesity and breast cancer in postmenopausal women. Cancer Res.

[75] Chew GL, Huang D, Huo CW, Blick T, Hill P, Cawson J (2013). Dynamic changes in high and low mammographic density human breast tissues maintained in murine tissue engineering chambers during various murine peripartum states and over time. Breast Cancer Res Treat.

[76] Chew GL, Huo CW, Huang D, Blick T, Hill P, Cawson J (2014). Effects of tamoxifen and oestrogen on histology and radiographic density in high and low mammographic density human breast tissues maintained in murine tissue engineering chambers. Breast Cancer Res Treat.

[77] Bertrand KA, Tamimi RM, Scott CG, Jensen MR, Pankratz VS, Visscher D (2013). Mammographic density and risk of breast cancer by age and tumor characteristics. Breast Cancer Res.

[78] Eriksson L, Czene K, Rosenberg L, Humphreys K, Hall P (2012). The influence of mammographic density on breast tumor characteristics. Breast Cancer Res Treat.

[79] Chumsri S, Howes T, Bao T, Sabnis G, Brodie A (2011). Aromatase, aromatase inhibitors, and breast cancer. J Steroid Biochem Mol Biol.

[80] Brown KA (2014). Impact of obesity on mammary gland inflammation and local estrogen production. J Mammary Gland Biol Neoplasia.

[81] Macaulay VM, Nicholls JE, Gledhill J, Rowlands MG, Dowsett M, Ashworth A (1994). Biological effects of stable overexpression of aromatase in human hormone-dependent breast cancer cells. Br J Cancer.

[82] Irahara N, Miyoshi Y, Taguchi T, Tamaki Y, Noguchi S (2006). Quantitative analysis of *aromatase*, *sulfatase* and *17β-HSD*1 mRNA expression in soft tissue metastases of breast cancer. Cancer Lett.

[83] Brodie A, Lu Q, Liu Y, Long B (1999). Aromatase inhibitors and their antitumor effects in model systems. Endocr Relat Cancer.

[84] Keshava N, Mandava U, Kirma N, Tekmal RR (2001). Acceleration of mammary neoplasia in aromatase transgenic mice by 7,12-dimethylbenz[*a*]anthracene. Cancer Lett.

[85] Ghosh S, Kang T, Wang H, Hu Y, Li R (2011). Mechanical phenotype is important for stromal aromatase expression. Steroids.

[86] Vachon CM, Sasano H, Ghosh K, Brandt KR, Watson DA, Reynolds C (2011). Aromatase immunoreactivity is increased in mammographically dense regions of the breast. Breast Cancer Res Treat.

[87] McInnes KJ, Brown KA, Knower KC, Chand AL, Clyne CD, Simpson ER (2008). Characterisation of aromatase expression in the human adipocyte cell line SGBS. Breast Cancer Res Treat.

[88] Simpson ER, Dowsett M (2002). Aromatase and its inhibitors: significance for breast cancer therapy. Recent Prog Horm Res.

[89] Cuzick J, Warwick J, Pinney E, Warren RM, Duffy SW (2004). Tamoxifen and breast density in women at increased risk of breast cancer. J Natl Cancer Inst.

[90] Vachon CM, Ingle JN, Suman VJ, Scott CG, Gottardt H, Olson JE (2007). Pilot study of the impact of letrozole vs. placebo on breast density in women completing 5 years of tamoxifen. Breast.

[91] Cigler T, Tu D, Yaffe MJ, Findlay B, Verma S, Johnston D (2010). A randomized, placebo-controlled trial (NCIC CTG MAP1) examining the effects of letrozole on mammographic breast density and other end organs in postmenopausal women. Breast Cancer Res Treat.

[92] Henry NL, Chan HP, Dantzer J, Goswami CP, Li L, Skaar TC (2013). Aromatase inhibitor-induced modulation of breast density: clinical and genetic effects. Br J Cancer.

[93] Cuzick J, Sestak I, Forbes JF, Dowsett M, Knox J, Cawthorn S (2014). Anastrozole for prevention of breast cancer in high-risk postmenopausal women (IBIS-II): an international, double-blind, randomised placebo-controlled trial. Lancet. 2014;383(9922):1041–8. A published erratum appears in. Lancet.

[94] Mantovani A, Allavena P, Sica A (2004). Tumour-associated macrophages as a prototypic type II polarised phagocyte population: role in tumour progression. Eur J Cancer.

[95] Van Ginderachter JA, Meerschaut S, Liu Y, Brys L, De Groeve K, Hassanzadeh Ghassabeh G (2006). Peroxisome proliferator-activated receptor γ (PPARγ) ligands reverse CTL suppression by alternatively activated (M2) macrophages in cancer. Blood.

[96] Madsen DH, Bugge TH (2013). Imaging collagen degradation in vivo highlights a key role for M2-polarized macrophages in extracellular matrix degradation. Oncoimmunology.

[97] Madsen DH, Leonard D, Masedunskas A, Moyer A, Jurgensen HJ, Peters DE (2013). M2-like macrophages are responsible for collagen degradation through a mannose receptor-mediated pathway. J Cell Biol.

[98] Kraus S, Arber N (2009). Inflammation and colorectal cancer. Curr Opin Pharmacol.

[99] Morris PG, Hudis CA, Giri D, Morrow M, Falcone DJ, Zhou XK (2011). Inflammation and increased aromatase expression occur in the breast tissue of obese women with breast cancer. Cancer Prev Res (Phila).

[100] Gottfried E, Kunz-Schughart LA, Weber A, Rehli M, Peuker A, Müller A (2008). Expression of CD68 in non-myeloid cell types. Scand J Immunol.

[101] Ansieau S, Courtois-Cox S, Morel AP, Puisieux A (2011). Failsafe program escape and EMT: a deleterious partnership. Semin Cancer Biol.

[102] Raviraj V, Zhang H, Chien HY, Cole L, Thompson EW, Soon L (2012). Dormant but migratory tumour cells in desmoplastic stroma of invasive ductal carcinomas. Clin Exp Metastasis.

[103] Burnet FM (1970). The concept of immunological surveillance. Prog Exp Tumor Res.

[104] Shankaran V, Ikeda H, Bruce AT, White JM, Swanson PE, Old LJ (2001). IFNγ and lymphocytes prevent primary tumour development and shape tumour immunogenicity. Nature.

[105] Vial T, Descotes J (2003). Immunosuppressive drugs and cancer. Toxicology.

[106] Mantovani A, Allavena P, Sica A, Balkwill F (2008). Cancer-related inflammation. Nature.

[107] Unsworth A, Anderson R, Britt K (2014). Stromal fibroblasts and the immune microenvironment: partners in mammary gland biology and pathology?. J Mammary Gland Biol Neoplasia.

[108] Hussein MR, Hassan HI (2006). Analysis of the mononuclear inflammatory cell infiltrate in the normal breast, benign proliferative breast disease, in situ and infiltrating ductal breast carcinomas: preliminary observations. J Clin Pathol.

[109] Reeves KW, Weissfeld JL, Modugno F, Diergaarde B (2011). Circulating levels of inflammatory markers and mammographic density among postmenopausal women. Breast Cancer Res Treat.

[110] Stone J, Willenberg L, Apicella C, Treloar S, Hopper J (2012). The association between mammographic density measures and aspirin or other NSAID use. Breast Cancer Res Treat.

[111] Degnim AC, Brahmbhatt RD, Radisky DC, Hoskin TL, Stallings-Mann M, Laudenschlager M (2014). Immune cell quantitation in normal breast tissue lobules with and without lobulitis. Breast Cancer Res Treat.

[112] Jindal S, Gao D, Bell P, Albrektsen G, Edgerton SM, Ambrosone CB (2014). Postpartum breast involution reveals regression of secretory lobules mediated by tissue-remodeling. Breast Cancer Res.

[113] Gierach GL, Loud JT, Chow CK, Prindiville SA, Eng-Wong J, Soballe PW (2010). Mammographic density does not differ between unaffected *BRCA1/2* mutation carriers and women at low-to-average risk of breast cancer. Breast Cancer Res Treat.

[114] Mitchell G, Antoniou AC, Warren R, Peock S, Brown J, Davies R (2006). Mammographic density and breast cancer risk in BRCA1 and BRCA2 mutation carriers. Cancer Res.

