# Supplementary figures and images for: High mammographic density is associated with an increase in stromal collagen and immune cells within the mammary epithelium
Source: Breast Cancer Res. 2015 Jun 4;17(1):79. doi: 10.1186/s13058-015-0592-1 (PMC4485361; doi:10.1186/s13058-015-0592-1)

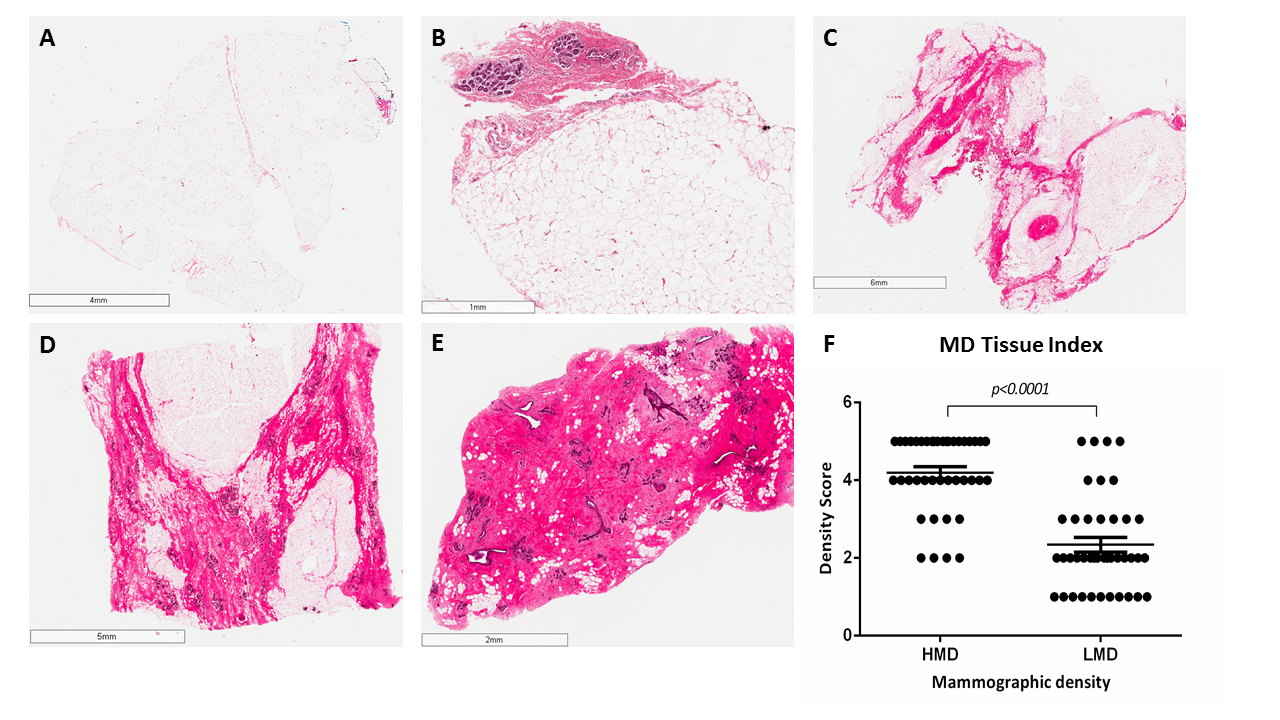

Supplement: Additional file 1: — MD semiquantitative assessment using the MD tissue index. Upon reviewing H&E-stained slides, tissue samples were assigned to one of the five categories: (1) 0 % to 10 % of tissue was stroma and/or epithelium/tissue section (A); (2) 11 % to 25 % of tissue was stroma and/or epithelium/tissue section (B); (3) 26 % to 50 % of tissue was stroma and/or epithelium/tissue section (C); (4) 51 % to 75 % of tissue was stroma and/or epithelium/tissue section (D); and (5) >75 % of tissue was stroma and/or epithelium/tissue section (E). (F) Scatterplot of assigned density category based on the percentage of stroma and epithelium per specimen shows that this categorical method of assessing MD produced results consistent with those derived from JMicroVision analyses as shown in Fig. 3 (n = 41 women). HMD high mammographic density, LMD low mammographic density. Owing to the different sizes of whole tissue sections, we show entire tissues, hence the inconsistencies of scales shown. [file 13058_2015_592_MOESM1_ESM.tif]

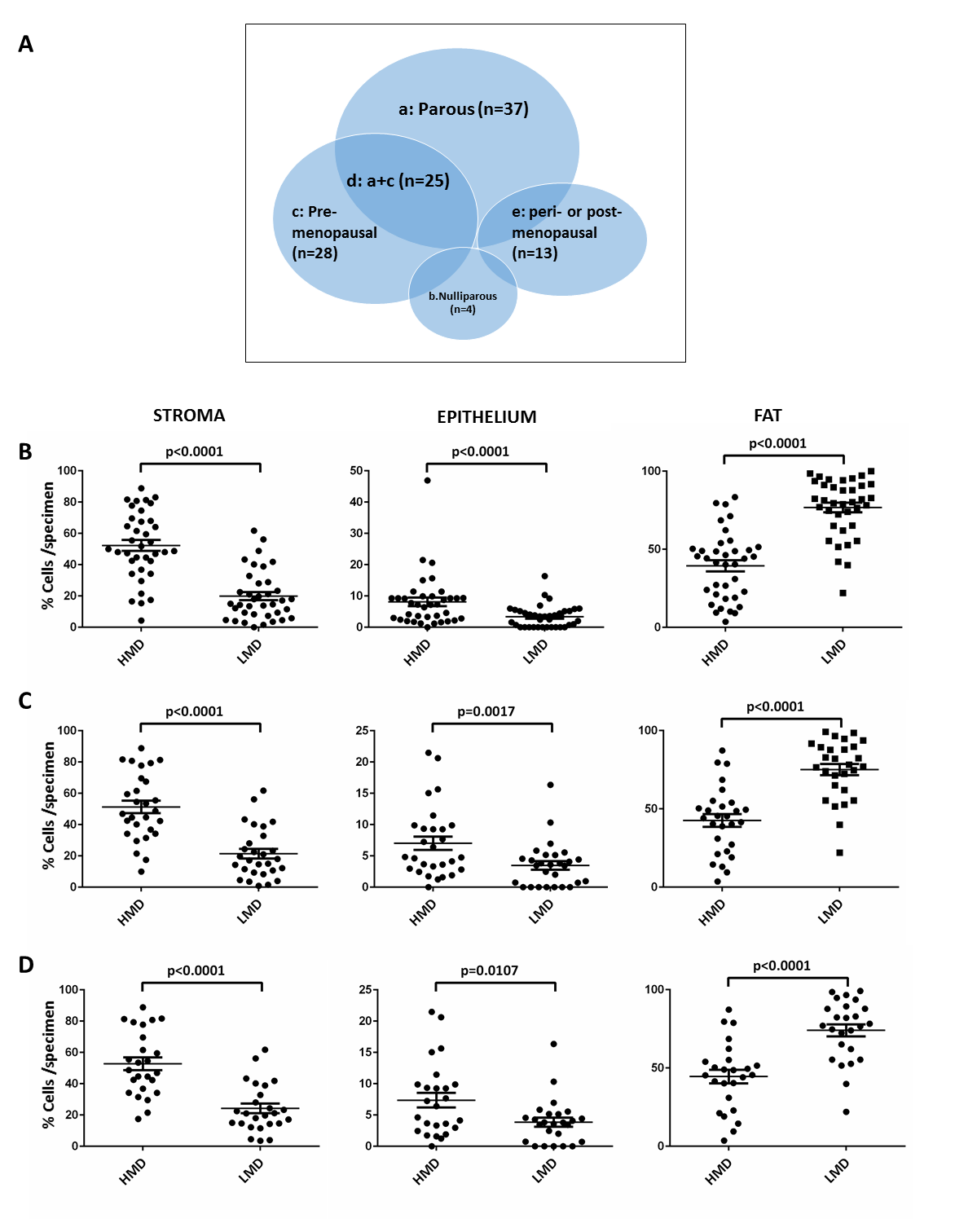

Supplement: Additional file 2: — Assessment of histological composition for parous and premenopausal women. (A) The distribution of participants by their parity and menopausal status. (B) The quantitative analyses by Wilcoxon matched-pairs signed-rank test of stroma, epithelium and fat percentages in premenopausal women [subset a (n = 37) shown in (A)]. (C) Quantitative analyses of stroma, epithelium and fat percentages in premenopausal women [subset b (n = 28) in (A)]. (D) Quantitative analyses of stroma, epithelium and fat percentages in parous and premenopausal women [subset d (n = 25) in (A)]. HMD high mammographic density, LMD low mammographic density. [file 13058_2015_592_MOESM2_ESM.tif]

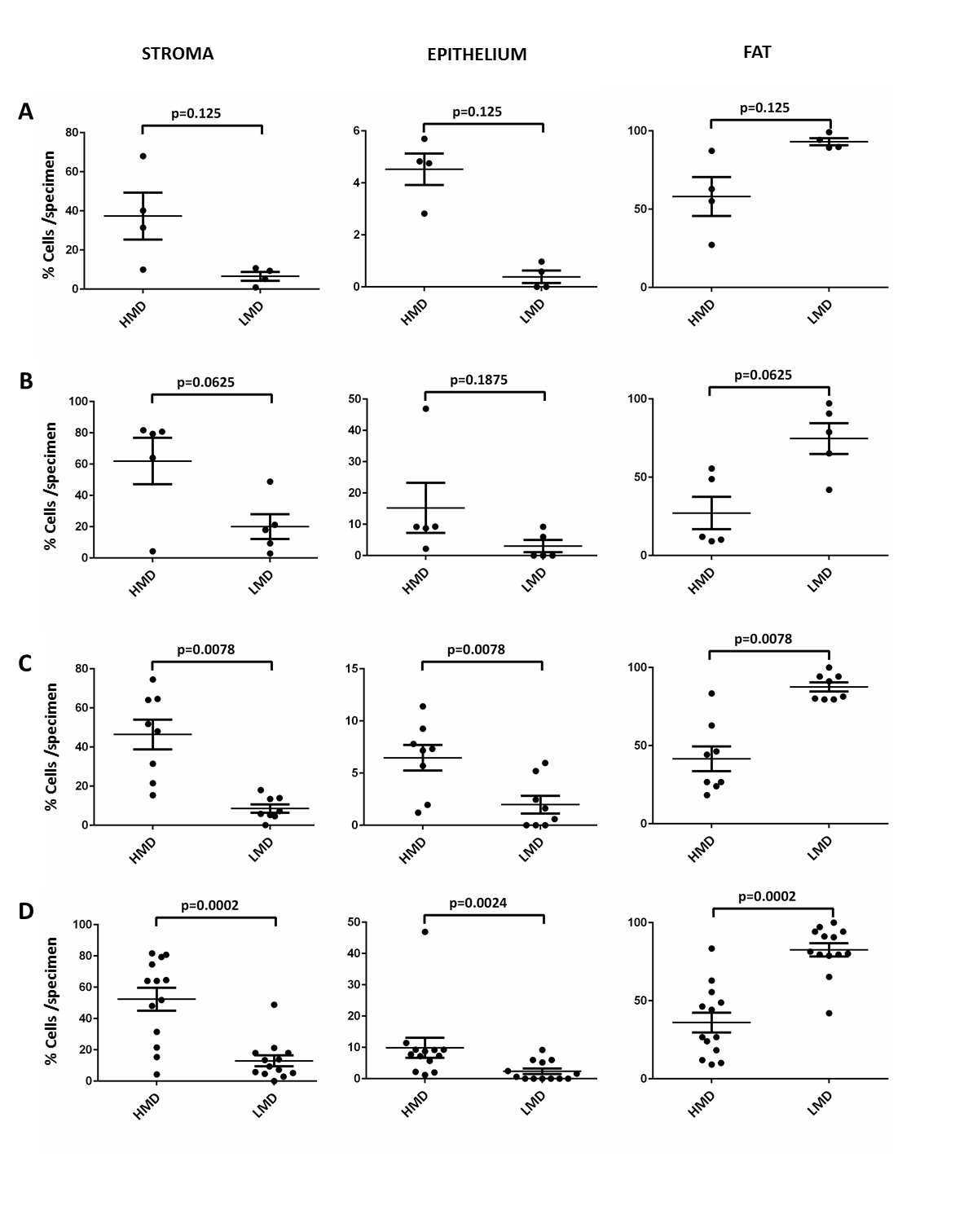

Supplement: Additional file 3: — Assessment of histological composition for nulliparous and peri- or postmenopausal women. Quantitative analyses by Wilcoxon matched-pairs signed rank test of the histological compositions of nulliparous women (n = 4) (A), perimenopausal women (n = 5) (B), postmenopausal women (n = 8) (C) and peri- and postmenopausal women (n = 13) (D). HMD high mammographic density, LMD low mammographic density. [file 13058_2015_592_MOESM3_ESM.tif]
